# TNF-α modulates cell proliferation via SOX4/TGF-β/Smad signaling in benign prostatic hyperplasia

**DOI:** 10.1038/s41419-025-07783-x

**Published:** 2025-07-01

**Authors:** Jinze Li, Bo Chen, Yin Huang, Xinyang Liao, Jia You, Zeyu Chen, Shu Ning, Asmaa Reda, Junwei Zhao, Biao Ran, Jingxing Bai, Mengli Zhu, Yan Wang, Hongying Chen, Qiang Wei, Dehong Cao, Liangren Liu

**Affiliations:** 1 Department of Urology, Institute of Urology, West China Hospital, Sichuan University, Chengdu, Sichuan China; 2 Department of Urology, People’s Hospital of Deyang City, Affiliated to Chengdu University of Traditional Chinese Medicine, Deyang, Sichuan China; 3 Ningbo Clinical Pathological Diagnosis Center, Ningbo, Zhejiang China; 4 Department of Urologic Surgery, School of Medicine, University of California Davis, Davis, CA USA; 5 Computational Biology and Bioinformatics, Zoology Department, Faculty of Science, Benha University, Banha, Egypt; 6 Department of Biochemistry and Molecular Medicine, UC Davis NCI-designated Comprehensive Cancer Center, University of California Davis, Sacramento, CA USA; 7 Core Facilities of West China Hospital, Sichuan University, Chengdu, Sichuan China

**Keywords:** Prostatic diseases, Cell signalling

## Abstract

Benign prostatic hyperplasia (BPH) is an age-related condition in men with a poorly defined etiology. Chronic inflammation is increasingly recognized as a key contributor to BPH progression; however, the underlying mechanisms remain incompletely understood. This study aimed to elucidate the role of a TNF-α-induced inflammatory microenvironment in regulating BPH progression. We demonstrated that TNF-α levels were significantly elevated in patients with BPH and positively correlated with key clinical characteristics. In vitro, TNF-α promoted the proliferation of prostatic cells. Mechanistically, TNF-α induced the overexpression of SOX4, which subsequently activated the TGF-β/Smad2/3 signaling axis, thereby enhancing cellular proliferation, promoting epithelial-mesenchymal transition (EMT), and exacerbating fibrosis. Importantly, metformin (Met) treatment reduced the expression levels of relevant inflammatory cytokines in the serum of BPH rats. Further analysis confirmed that Met inhibited the TGF-β/Smad2/3 signaling pathway by downregulating the expression of SOX4, thus suppressing cell proliferation, reversing EMT, alleviating fibrosis, and ultimately exerting anti-BPH effects. Collectively, our findings suggest that TNF-α promotes BPH progression via activation of the SOX4/TGF-β/Smad2/3 axis, while Met exerts therapeutic effects by targeting this pathway. These results highlight SOX4 as a potential therapeutic target for BPH and support the clinical potential of Met in BPH management.

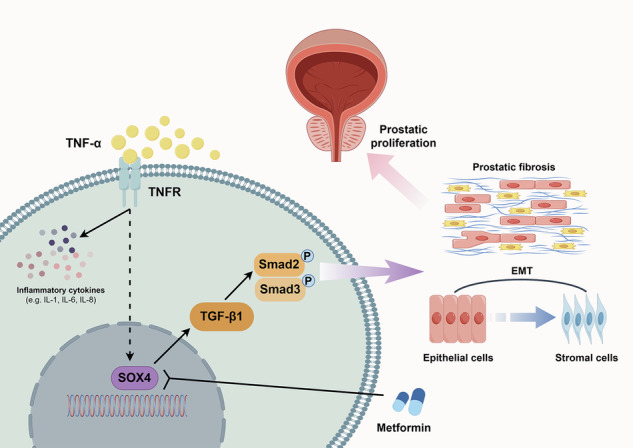

## Introduction

Benign prostatic hyperplasia (BPH) is a prevalent condition in aging male, and its prevalence increases with age [1, 2]. BPH is histologically characterized by epithelial and stromal hyperplasia within the periurethral and transition zones of the prostate gland, leading to bladder outlet obstruction and associated lower urinary tract symptoms (LUTS), which substantially impair the quality of life of elderly men [3, 4]. Although its pathogenesis has been extensively investigated, involving factors such as sex hormone imbalance, tissue remodeling, metabolic dysregulation, and immune inflammation [5–8], the precise molecular mechanisms underlying BPH development and its progression to symptomatic LUTS remain incompletely understood.

Recently, the relationship between chronic inflammation and BPH has garnered increasing attention. Evidence from our previous study and others indicates that inflammatory infiltrations, including lymphocytes, macrophages, and cytokines, are frequently observed in BPH tissues [9–12]. Moreover, our preliminary research revealed that chronic inflammation in patients with BPH is a potential cause of more severe LUTS and a greater likelihood of acute urinary retention (AUR) [13]. Chronic inflammation has been shown to regulate epithelial-mesenchymal transition (EMT), fibrosis, and proliferation in the prostate [1, 14, 15], suggesting that inflammation may play a crucial role in the occurrence and progression of BPH. However, the detailed mechanism by which inflammation leads to prostatic hyperplasia had not yet been fully elucidated.

Tumor necrosis factor-alpha (TNF-α) is a pleiotropic cytokine produced by immune cells and is notable for its potent proinflammatory effects [16]. It can activate downstream signaling pathways by binding to TNFR1 and/or TNFR2, thereby mediating various biological responses, including cell proliferation, apoptosis, and inflammation [17]. Studies have indicated that the upregulation of TNF-α expression could modulate the inflammatory environment in BPH and promote the proliferation of prostate stromal cells [18]. Additionally, the use of TNF-α antagonists has been shown to reduce epithelial hyperplasia, NF-κB activation, and macrophage-mediated inflammation within prostate tissue [19]. These findings suggest that TNF-α is involved in the initiation and progression of BPH, though its exact role in BPH pathology remains unclear.

SRY-related high-mobility-group box 4 (SOX4), a member of the SOX family of transcription factors, plays key roles in regulating cell fate determination, proliferation, and differentiation [20, 21]. SOX4 has been shown to be upregulated in various cancers, including colorectal, breast, and prostate cancer, where it promotes tumorigenesis and progression by activating critical signaling pathways such as transforming growth factor-beta (TGF-β) and Wnt/β-catenin [22–24]. Interestingly, SOX4 is also considered a significant molecular target in chronic inflammatory diseases. Studies have shown that SOX4 is highly expressed in the synovium of arthritis patients and serves as a target and key mediator of TNF-α during the transformation of fibroblast-like synoviocytes (FLS) [25, 26]. However, there are currently no reports on the expression and function of SOX4 in BPH.

Notably, recent studies have highlighted the role of TNF-α in regulating the expression and activity of key transcription factors in the pathogenesis of inflammatory diseases [26, 27]. To explore potential therapeutic strategies for BPH, we further investigated the application of metformin (Met). Met, a widely used hypoglycemic agent, has been demonstrated to exert anti-inflammatory, anti-proliferative and anti-fibrotic effects by inhibiting the TGF-β and NF-κB signaling pathways [28, 29]. Moreover, previous studies have demonstrated that Met suppresses cell proliferation by downregulating SOX4 expression, thereby exerting antiproliferative effects [30]. Therefore, this present study aimed to elucidate the role of TNF-α in shaping the inflammatory microenvironment of BPH and its regulatory influence on SOX4 expression, as well as to further explore the therapeutic potential of Met in ameliorating BPH through SOX4 inhibition, thereby offering new mechanistic insights into BPH pathogenesis and intervention.

## Materials and methods

### Human samples and clinical data

Normal and BPH blood samples were obtained from healthy male subjects undergoing physical examinations at West China Hospital and from BPH patients undergoing transurethral resection of the prostate, respectively. Prostatic hyperplasia tissues were collected from BPH patients undergoing surgery, and confirmed as BPH by postoperative pathological examination. Normal prostate tissues were obtained from male patients under 40 years of age who underwent radical cystectomy, and pathological examination by two independent pathologists revealed no hyperplasia. The relevant clinical data of the collected patients were recorded. All experiments involving human specimens were approved by the Ethics Review Committee of West China Hospital, Sichuan University, and informed consent was obtained from all patients. The approval number for handling human subjects was 2022-1700.

### Animal model

Eight-week-old male Sprague-Dawley rats (250–300 g) were purchased from SPF Biotechnology Co. Ltd (Beijing, China). After a one-week acclimatization period, the rats were randomly divided into three groups (*n* = 5 per group). The control group underwent a sham operation, while the other two groups were castrated to eliminate the effects of intrinsic testosterone: Control group: non-pathological prostate, and was subcutaneously administered corn oil; BPH group: subcutaneously administered testosterone propionate (TP) (dissolved in corn oil) at 4 mg/kg/day after castration; BPH + Met group: subcutaneously administered TP at 4 mg/kg/day after castration + orally administered Met at 500 mg/kg/day. All rats were allowed to recover for one week after surgery, and the treatment duration was 4 weeks (28 days). The dose of Met was determined on the basis of previous studies [31]. Serum and prostate tissues were collected 24 h after the last treatment. The serum from all the rats was centrifuged and stored at –80 °C, and the prostate tissues were weighed and either fixed in 4% paraformaldehyde or preserved in liquid nitrogen. This study was approved by the Animal Ethics Review Committee of West China Hospital, Sichuan University. The approval number of the animal experiments was 20230308039. All animal experiments were conducted in strict accordance with the National Institutes of Health (NIH) guidelines for the Care and Use of Laboratory Animals.

### Cell culture and treatment

The human normal prostate stromal cell line (WPMY-1) was purchased from the American Type Culture Collection (ATCC; Manassas, VA, USA) and cultured in DMEM medium (Thermo, MA, USA) supplemented with 5% fetal bovine serum (FBS; Gibco, Australia), and 1% penicillin-streptomycin (HyClone, MA, USA). The human BPH epithelial cell line (BPH-1) was purchased from Servicebio Co., Ltd (Wuhan, China) and cultured in RPMI-1640 medium (Thermo, MA, USA) containing 10% FBS, and 1% penicillin-streptomycin. The cells were treated with TNF-α (Novoprotein, China) or Met (Selleck, China) according to the experimental requirements. TNF-α and Met were both dissolved in normal saline and directly added to the culture medium at the beginning of the treatment period, without daily replenishment. The culture medium was not changed during the treatment, which lasted for 0, 24, 48, or 72 h.

### Cell virus infection and plasmid transfection

In this study, SOX4 knockdown was achieved by transducing lentiviruses containing short hairpin RNAs (shRNAs) targeting SOX4 (Sh-SOX4; Genechem, Shanghai, China) (Table S1), and two shRNA sequences with better inhibition efficiency were selected for further research. Nontargeting shRNA (Sh-NC) was used as a negative control (Table S1). A SOX4 overexpression plasmid (OE-SOX4; Genechem, Shanghai, China) or an empty plasmid (Vector) was transfected into cells via Lipofectamine 3000 transfection reagent (Invitrogen, CA, USA) to achieve SOX4 overexpression.

### Cell viability assessment

In this study, cell viability was determined via the Cell Counting Kit-8 (CCK-8) assay (Dojindo, Rockville, USA) and EdU assay (Elabscience, Wuhan, China). WPMY-1 cells (3 × 10³ cells/well) or BPH-1 cells (3 × 10³ cells/well) were seeded into 96-well culture plates and treated or transduced for 0, 24, 48, or 72 h. CCK8 experiment: The medium in each well was replaced with 100 μL of fresh complete medium containing 10 μL of CCK-8 solution. Finally, the cells were incubated in the dark at 37 °C for 2 h, and the absorbance at 450 nm was measured via an Eon™ microplate reader (Bio-Tek, VT, USA). EdU experiment: Cells in each well were first incubated with EdU solution (50 μM/well) for 2 h; followed by fixing the cells with 4% paraformaldehyde for 15 min; and then cells were washed with 0.5% TritonX-100 for 20 min. Next, cells were incubated with Click reaction solution for 30 min; then, cells were washed with PBS for 10 min. Finally, cells were incubated with DAPI solution for 10 min at room temperature. Photographs were taken for observation using a laser confocal fluorescence microscope (ZEISS, Oberkochen, Germany).

### Flow cytometry analysis

The cells were inoculated in 6-well plates at a concentration of 2 × 10^5^ cells/well, and cells were processed and collected according to experimental requirements. Cell cycle analysis was performed according to the instructions provided in the Cell Cycle Detection Kit (KeyGen, Jiangsu, China). Cells were fixed in 500 μL of 75% ethanol at 4 °C overnight, washed, filtered, and then stained with PI/RNase dye working solution. After incubating at room temperature in the dark for 30 min, the cell cycle was detected and recorded using a CytoFLEX flow cytometer (Beckman, IN, USA). Cell apoptosis was directly detected using the Annexin V-APC/PI apoptosis kit (Elabscience, Wuhan, China) as per the manufacturer’s manual. Cells were resuspended in 500 μL of 1×binding buffer, incubated with 5 μL of Annexin V-APC and 5 μL of PI Reagent at room temperature in the dark for 15–20 min, and then apoptosis was evaluated using the flow cytometer.

### RNA sequencing (RNA-seq) and bioinformatics analysis

We performed RNA sequencing on WPMY-1 cells treated with or without TNF-α and with or without SOX4 knockdown. The transcriptome analysis was outsourced to Novogene (Beijing, China), Briefly, RNA was extracted using TRIzol (Invitrogen, CA, USA), followed by quantification and qualification of RNA samples. Sequencing libraries were then prepared using the NEBNext® Ultra™ RNA Library Prep Kit for Illumina® (NEB, USA). After quantifying the sequenced genes to obtain their Read counts, differentially expressed genes (DEGs) was performed using the R package “DESeq2”. Gene Ontology (GO) enrichment analysis and Kyoto Encyclopedia of Genes and Genomes (KEGG) enrichment analysis were conducted using the R package “clusterProfiler”. We downloaded RNA-seq raw data related to BPH from Gene Expression Omnibus (GEO) database, specifically GSE119195, GSE132714, and GSE167196. All DEGs was analyzed by using R software (4.1.1).

### RNA extraction and real-time quantitative polymerase chain reaction (RT-qPCR)

Total RNA was extracted using the cell Total RNA Isolation Kit (FOREGENE, Chengdu, China) according to the manufacturer’s instructions. The total RNA was then reverse transcribed into complementary DNA (cDNA) using the RevertAid First Strand cDNA Synthesis Kit (Thermo, MA, USA). As previously described, qPCR was performed using the SYBR Green PCR Kit (FOREGENE, Chengdu, China) on a CFX96 Touch Real-Time PCR Detection System (Bio-Rad, CA, USA). The housekeeping gene GAPDH was used for normalization, and the relative gene expression levels were determined using the 2^−ΔΔCt^ method. Primer sequences are listed in Table S2.

### Western blot (WB) analysis

Total protein was extracted using RIPA lysis buffer (Thermo, MA, USA) containing protease inhibitor and phosphatase inhibitor (M5293 and M7528, AbMole, TX, USA) on ice for 30 min. After separating the proteins by sodium dodecyl sulfate-polyacrylamide gel electrophoresis (SDS-PAGE; Epizyme, Shanghai, China), the gel was transferred onto a polyvinylidene fluoride (PVDF) membrane (Millipore, MA, USA). The membrane was incubated with 5% bovine serum albumin (BSA) solution for 2 h, followed by incubation with diluted primary antibodies at 4 °C overnight. After several washes, the membrane was incubated with secondary antibodies at room temperature for 1 h according to the primary antibody source. The antigen-antibody reaction was detected using an enhanced chemiluminescence (ECL) detection kit (Millipore, MA, USA). The target protein bands were visualized using the ChemiDoc MP Imager System (Bio-Rad, CA, USA). Finally, ImageJ software was used to quantify and normalize the target protein bands. For protein expression analysis, target protein expression was normalized to Vinculin or GAPDH, and results were expressed relative to the control group, which was set to 1 for quantification purposes. The primary antibodies and their corresponding information are summarized in Table S3.

### Cell immunofluorescence (IF)

The cells were inoculated in 24-well plates at a concentration of 2 × 10^5^ cells/well. Then, cells were fixed with 4% paraformaldehyde for 20 min, permeabilized with 0.1% Triton X-100 for 5 min, and incubated with 5% goat serum at 37 °C for 30 min. The cells were then incubated with DAPI stain for 10 min, and finally visualized using a laser confocal fluorescence microscope (ZEISS, Oberkochen, Germany).

### Enzyme-linked immunosorbent assay (ELISA) and clinical correlation analysis

The serum levels of TNF-α, IL-1β, and IL-6 were measured using ELISA kit (Bioswamp, Wuhan, China) according to the manufacturer’s protocol. Absorbance at 450 nm was recorded in a microplate reader, and calibration curves were constructed based on the absorbance and concentrations of each standard. The sample concentrations were then determined from the calibration curves. Pearson correlation analysis was performed to explore the relationship between the TNF-α levels and clinical characteristics of BPH. Additionally, correlations between serum TNF-α concentration and inflammatory factors IL-1β and IL-6 were analyzed.

### Histological staining

Tissues were fixed in 4% paraformaldehyde and embedded in paraffin. The tissues were then sectioned into 4-μm-thick slices. Subsequently, we stained the sections with hematoxylin and eosin (HE) for evaluating histopathological features, and with Masson’s trichrome stain for assessing collagen fiber deposition. Imaging of all stained sections was performed using an optical microscope (Olympus, Tokyo, Japan). Additionally, ImageJ software was utilized to quantify the area of collagen fiber-positive in the Masson-stained sections.

### Immunohistochemistry (IHC)

For IHC analysis, tissue sections were deparaffinized and hydrated, followed by overnight incubation with primary antibodies at 4 °C. After incubation with corresponding secondary antibodies, sections were stained with diaminobenzidine (DAB) and counterstained with Mayer’s hematoxylin. Immunoreactive scores for IHC staining images were evaluated using the IHC Profiler plugin based on ImageJ software [32]. The analysis combines the average gray value of positive cells (staining intensity) and the percentage of positive area (staining area) as a measure, and finally gives four results, high positive (4 points), positive (3 points), low positive (2 points) and negative (1 point). The primary antibody details, including sources and dilutions, are summarized in Table S4.

### Statistical analysis

The data are presented as the mean ± standard deviation (SD) from at least three independent experiments. Student’s *t*-test and one-way ANOVA were used to assess differences between two groups and among multiple groups, respectively. Statistical analyses and graphical plotting for the study were performed using GraphPad Prism 9.0. *P* < 0.05 was considered statistically significant and denoted as follows: *P < 0.05, **P < 0.01, ***P < 0.001, ****P < 0.0001.

## Results

### TNF-α is upregulated in BPH patients and promotes the proliferation of BPH cells as well as the inflammatory response

**Fig. 1:**
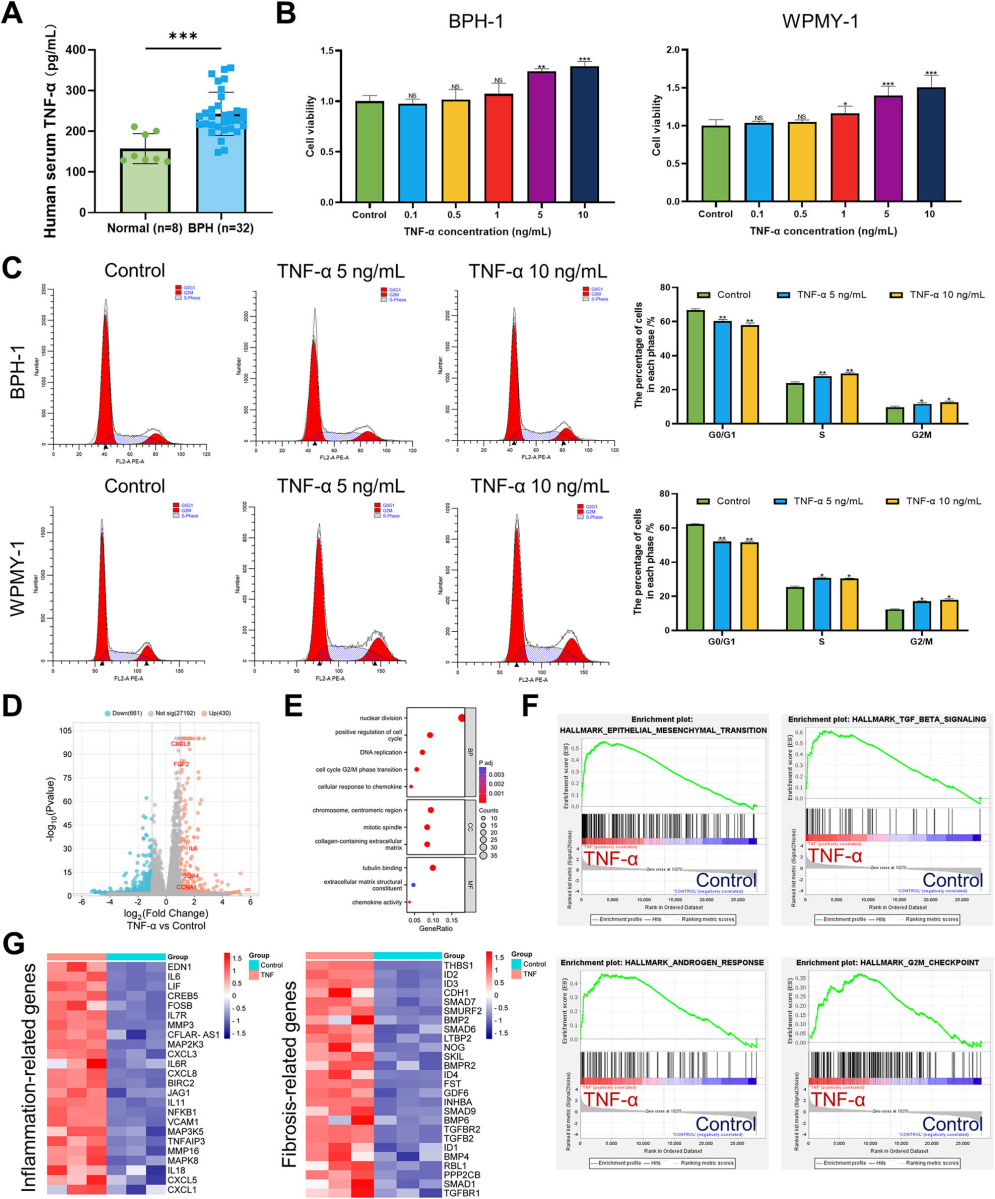
TNF-α is upregulated in human BPH and promotes the proliferation of BPH-1 and WPMY-1 cells. **A** TNF-α is significantly upregulated in BPH patients’ serum. **B** BPH-1 and WPMY-1 cells were plated in 96-well plates (3 × 10^3^ cells/well) overnight. Then, BPH-1 and WPMY-1 cells were treated with doses of TNF-α (0, 0.1, 0.5, 1, 5, and 10 ng/mL) for 3 days. Cells viability was determined by CCK-8 test. **C** BPH-1 and WPMY-1 cells were plated in 6-well plates (2 × 10^6^ cells/well) overnight. Then, BPH-1 and WPMY-1 cells treated with TNF-α (0, 5, and 10 ng/mL) for 3 days, and harvested for cell cycle test via flow cytometry. **D** Valcano plot showing the differentiated expressed genes in WPMY-1 cells treated with TNF-α. **E** GO analysis showing pathways upregulated in WPMY-1 cells treated with TNF-α. **F** Enrichment plots of GSEA analyses for pathways in TNF-α group compared with control group. **G** Heatmap clustering the upregulated genes related to inflammation and fibrosis in WPMY-1 cells. Data are expressed as the means ± SEMs (*p < 0.05, **p < 0.01, ***p < 0.001, ns: not significant).

First, we measured the expression of TNF-α in the serum of BPH patients and normal individuals, and the results revealed that the serum TNF-α levels in BPH patients were significantly greater than those in normal individuals (Fig. 1A). Correlation analysis revealed a positive correlation between TNF-α expression and prostate volume (*r* = 0.55, *P* = 0.001) as well as the International Prostate Symptom Score (IPSS) (*r* = 0.52, *P* = 0.002) in BPH patients (Fig. S1A and Tables S5-6). However, no statistical correlation was detected between TNF-α expression and prostate-specific antigen levels, maximum flow rate, or post-void residual (Table S6). To investigate the function of TNF-α in BPH, a TNF-α-induced inflammatory cell model was constructed. The CCK8 assay results demonstrated that TNF-α modulated the viability of WPMY-1 and BPH-1 cells in a dose-dependent manner and significantly promoted the proliferation of BPH cells at concentrations of 5 ng/mL and 10 ng/mL (Fig. 1B). Moreover, EdU experiments further confirmed that TNF-α could promote the proliferative viability of WPMY-1 cells (Fig. S1B). Flow cytometry revealed that under TNF-α stimulation, the proportion of cells in the G0/G1 phase decreased, whereas the proportions in the G2/M and S phases increased (Fig. 1C). To further assess the role of TNF-α, RNA-seq analysis was performed on TNF-α-induced WPMY-1 cells. The results revealed many DEGs between the TNF-α-induced group and the control group, with 430 genes significantly upregulated and 661 genes significantly downregulated (Fig. 1D). Subsequent GO analysis indicated that TNF-α induction primarily involved nuclear division, positive regulation of cell cycle, DNA replication, cell cycle G2/M phase transition, collagen-containing extracellular matrix, tubulin binding, and chemokine activity (Fig. 1E). Additionally, GSEA enrichment analysis found that TNF-α significantly promoted the upregulation of EMT, TGF-β signaling, androgen response, G2/M checkpoint pathways, and cell population proliferation (Figs. 1F and S1C). Heatmap analysis also revealed upregulation of genes related to inflammation, Fibrosis, cell proliferation, and cell cycle following TNF-α treatment (Figs. 1G and S1D). Notably, GO BP analysis also indicated that immune and inflammatory response-related pathways, including T cell differentiation, positive regulation of T cell differentiation, and response to interleukin-1, were upregulated in the TNF-α-treated group compared with the control group (Fig. S2A). GSEA analysis revealed that three inflammatory response pathways were upregulated in the TNF-α-treated group, including TNF signaling via NF-κB, inflammatory response, and IL6-JAK-STAT3 signaling (Fig. S2B). Additionally, RT-qPCR analysis revealed that, compared to the control, TNF-α significantly promoted the upregulation of five inflammatory molecules, including IL-1α, IL-1β, IL-6, IL-8, and IL-18 (Fig. S2C, D). In summary, our data suggest that TNF-α is highly expressed in BPH patients and promotes the proliferation and inflammatory response of BPH cells.

### TNF-α regulates SOX4/TGF-β/Smad, EMT, and fibrosis related gene programs in BPH cells

**Fig. 2:**
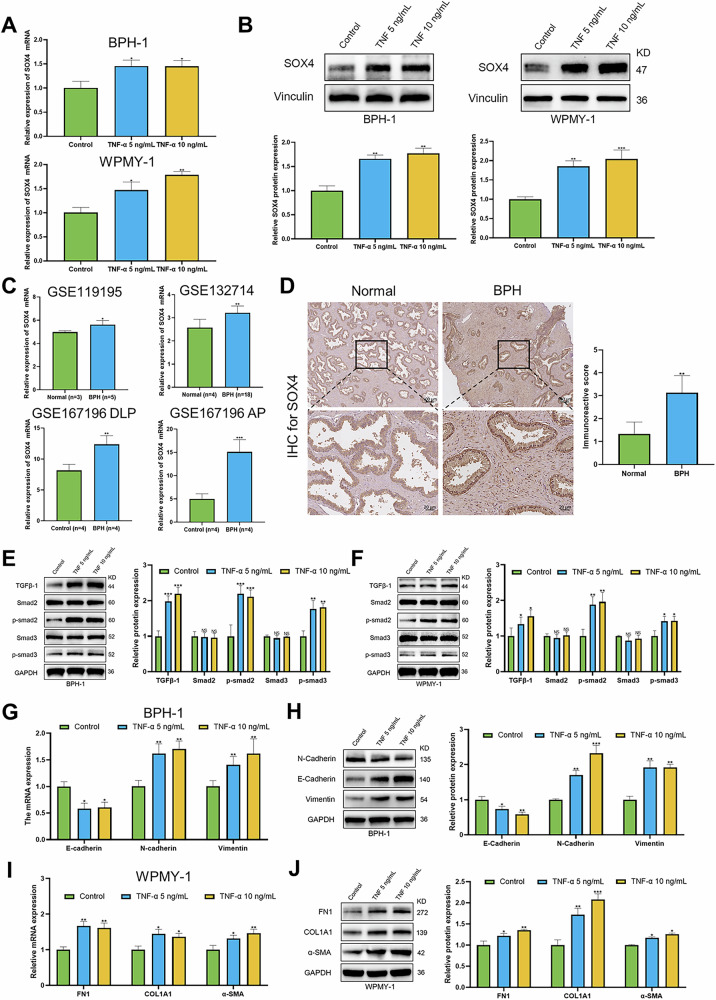
TNF-α induces SOX4 expression and activates the TGF-β/Smad signaling pathway and its downstream genes. **A** RT-PCR was used to test the mRNA levels of SOX4 in WPMY-1 and BPH-1 cells treated with TNF-α. **B** WB was used to test the protein levels of SOX4 in WPMY-1 and BPH-1 cells treated with TNF-α. **C** GEO data showing SOX4 expression in BPH samples from GSE119195, GSE132714, and GSE167196. **D** Representative SOX4 IHC staining and quantification in normal and BPH prostate samples. **E**, **F** WB analysis of TGF-β/Smad signaling protein expression in BPH-1 and WPMY-1 cells treated with TNF-α (5 ng/mL and 10 ng/mL) for 3 days. **G**, **H** RT-PCR and WB analysis of the mRNA and protein expression of EMT markers in BPH-1 cells treated with TNF-α (5 ng/mL and 10 ng/mL) for 3 days. **I**, **J** RT-PCR and WB analysis of the mRNA and protein expression of fibrosis markers in WPMY-1 cells treated with TNF-α (5 and 10 ng/ml) for 3 days. Data are expressed as the means ± SEMs (*p < 0.05, **p < 0.01, ***p < 0.001, ns: not significant).

The RNA-seq data from this study indicated that the mRNA level of SOX4 in the TNF-α treated group was significantly higher than in the control group. Moreover, RT-qPCR and WB confirmed that TNF-α induced the upregulation of SOX4 expression in BPH-1 and WPMY-1 cells (Fig. 2A, B). Furthermore, analysis of publicly available RNA-seq datasets (GSE119195, GSE132714, and GSE167196) revealed that the mRNA levels of SOX4 were significantly upregulated in the BPH group than in the normal group (Fig. 2C). We performed IHC staining on BPH and normal prostate tissues, and the results revealed that SOX4 was highly expressed in hyperplastic prostate tissues, with expression observed in both the epithelial and stromal components of the prostate tissue (Fig. 2D). The IF staining further revealed that SOX4 was expressed in both epithelial and stromal cells, and was located in the nucleus and cytoplasm (Fig. S2E). In light of the GSEA enrichment analysis results from the aforementioned RNA-seq data, we further investigated the effects of TNF-α on the TGF-β signaling pathway. The protein expression levels of five TGF-β/Smad signaling biomarkers, such as TGF-β1, Smad2, p-smad2, Smad3, and p-smad3, were assessed in TNF-α-treated BPH-1 and WPMY-1 cells. The results indicated that TNF-α treatment significantly upregulated the expression of TGF-β1, p-smad2, and p-smad3, and the p-smad2/Smad2 and p-smad3/Smad3 ratios were also elevated (Fig. 2E, F). It is noteworthy that TNF-α significantly altered the expression of EMT-associated markers in BPH-1 cells, including the downregulation of the epithelial marker E-cadherin and the upregulation of mesenchymal markers N-cadherin and Vimentin (Fig. 2G, H). Additionally, it also induced the expression of fibrosis markers in WPMY-1 cells, including elevated levels of FN1, COL1A1, and α-SMA (Fig. 2I, J). To sum up, this study demonstrates that TNF-α promotes the upregulation of SOX4 and activates the TGF-β1/Smad2/3 signaling pathway, which in turn induces the cellular EMT process and exacerbates fibrosis.

### Effects of SOX4 knockdown on BPH cells

**Fig. 3:**
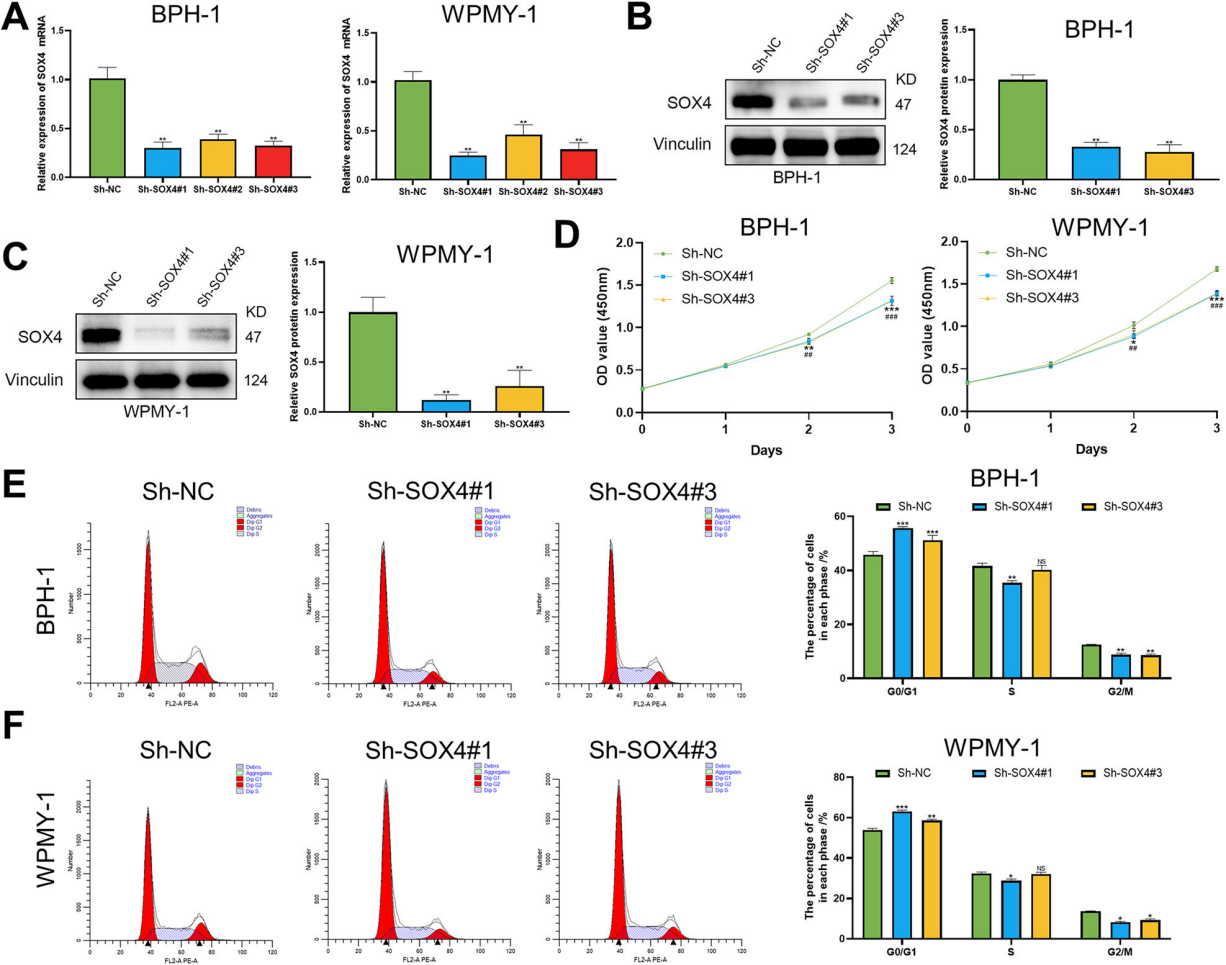
Effects of SOX4 knockdown on cell viability and cell cycle in BPH-1 and WPMY-1 cells. **A**–**C** RT-PCR and WB were used to confirm the SOX4 knockdown effect in BPH-1 and WPMY-1 cells infected with Sh-NC or Sh-SOX4 lentivirus for 3 days. **D** BPH-1 and WPMY-1 cells were plated in 96-well plates (3 × 10^3^ cells/well) and infected with Sh-NC or Sh-SOX4 lentivirus for 0–3 days. Cell proliferation viability was determined by CCK-8 test. **E**, **F** Flow cytometry was used to test the cells cycle of BPH-1 and WPMY-1 cells infected with Sh-NC or Sh-SOX4 lentivirus for 3 days, retrospectively. Data are expressed as the means ± SEMs (*p < 0.05, **p < 0.01, ***p < 0.001, ns: not significant).

To evaluate the biological function of SOX4, we employed three different Sh-RNAs to knock down SOX4 in BPH-1 and WPMY-1 cells, respectively. The results showed that all three Sh-RNAs significantly reduced the mRNA levels of SOX4, with Sh-SOX4#1 and Sh-SOX4#3 demonstrating greater knockdown efficiency compared to Sh-SOX4#2 (Fig. 3A). Additionally, both Sh-RNAs significantly silenced the protein expression of SOX4 (Fig. 3B, C). Then, we found that SOX4 knockdown significantly inhibited the proliferation of BPH-1 cells, with a similar inhibitory effect observed in WPMY-1 cells (Figs. 3D and S3A). Flow cytometry analysis revealed SOX4 knockdown led to a marked increase in the proportion of cells in the G0/G1 phase, while the proportion of cells in G2/M and S phases was significantly decreased (Fig. 3E, F). Interestingly, this study found that SOX4 knockdown increased the apoptosis rate (Fig. S3B, C), with a decrease in the Bcl2 protein level and an increase in the Bax protein level (Fig. S3D). Additionally, WB experiments confirmed that SOX4 knockdown significantly reduced the expression of cell cycle-related proteins, including CDK2, CDK4, CDK6, Cyclin A1/A2, Cyclin D1, and Cyclin E2 (Fig. S3E, F).

### Effects of SOX4 overexpression on BPH cells

**Fig. 4:**
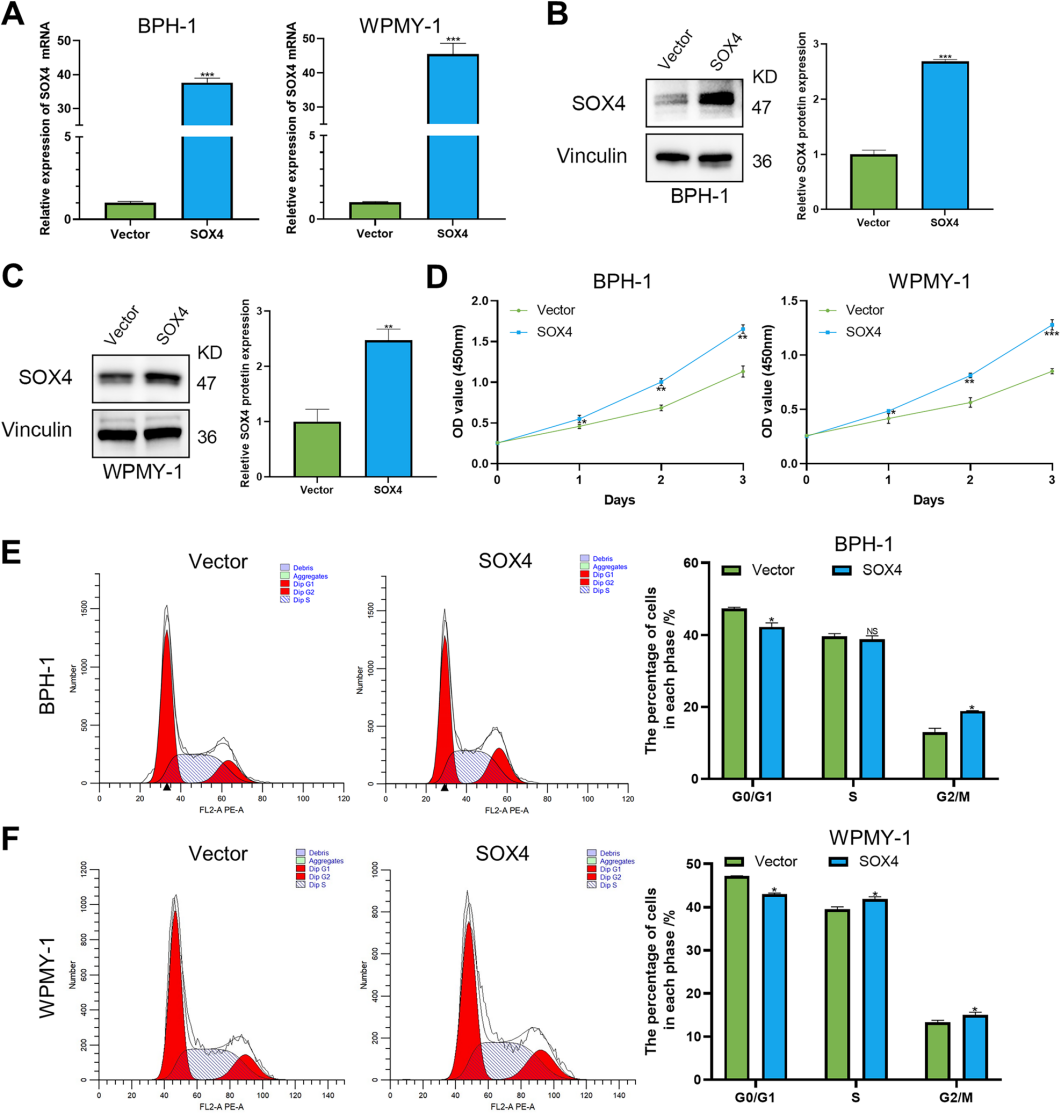
Overexpression of SOX4 regulates cell viability and cell cycle in BPH-1 and WPMY-1 Cells. **A**–**C** RT-PCR and WB were used to confirm the SOX4 overexpression effect in BPH-1 and WPMY-1 cells infected with vector or SOX4 lentivirus for 3 days. **D** BPH-1 and WPMY-1 cells were plated in 96-well plates (3 × 10^3^ cells/well) and infected with vector or SOX4 lentivirus for 0–3 days. Cell proliferation viability was determined by CCK-8 test, respectively. **E**, **F** Flow cytometry was used to test the cells cycle of BPH-1 and WPMY-1 cells infected with vector or SOX4 lentivirus for 3 days. Data are expressed as the means ± SEMs (*p < 0.05, **p < 0.01, ***p < 0.001, ns not significant).

We conducted SOX4 overexpression experiments in BPH-1 and WPMY-1 cells, and the results revealed that both the mRNA and protein levels of SOX4 were significantly increased (Fig. 4A–C). The CCK8 and EdU experiments confirmed that SOX4 overexpression significantly promoted the proliferation of BPH cells (Figs. 4D and S4A). In BPH-1 and WPMY-1 cells, SOX4 overexpression led to a significant decrease in the proportion of cells in the G0/G1 phase, while the proportion of cells in the G2/M phase increased (Fig. 4E, F). Furthermore, SOX4 overexpression significantly inhibited the apoptosis rate, resulting in an upregulation of Bcl2 protein expression and a downregulation of Bax expression (Fig. S4B–E). We also observed that SOX4 overexpression significantly increased the expression of CDK2, CDK4, CDK6, Cyclin A1/A2, Cyclin D1, and Cyclin E2 (Fig. S4F, G). These data confirm that SOX4 modulates prostate cell proliferation and may serve as a potential therapeutic target for BPH.

### SOX4 regulates EMT and fibrosis-related gene expression via the TGF-β1/Smad signaling pathway

**Fig. 5:**
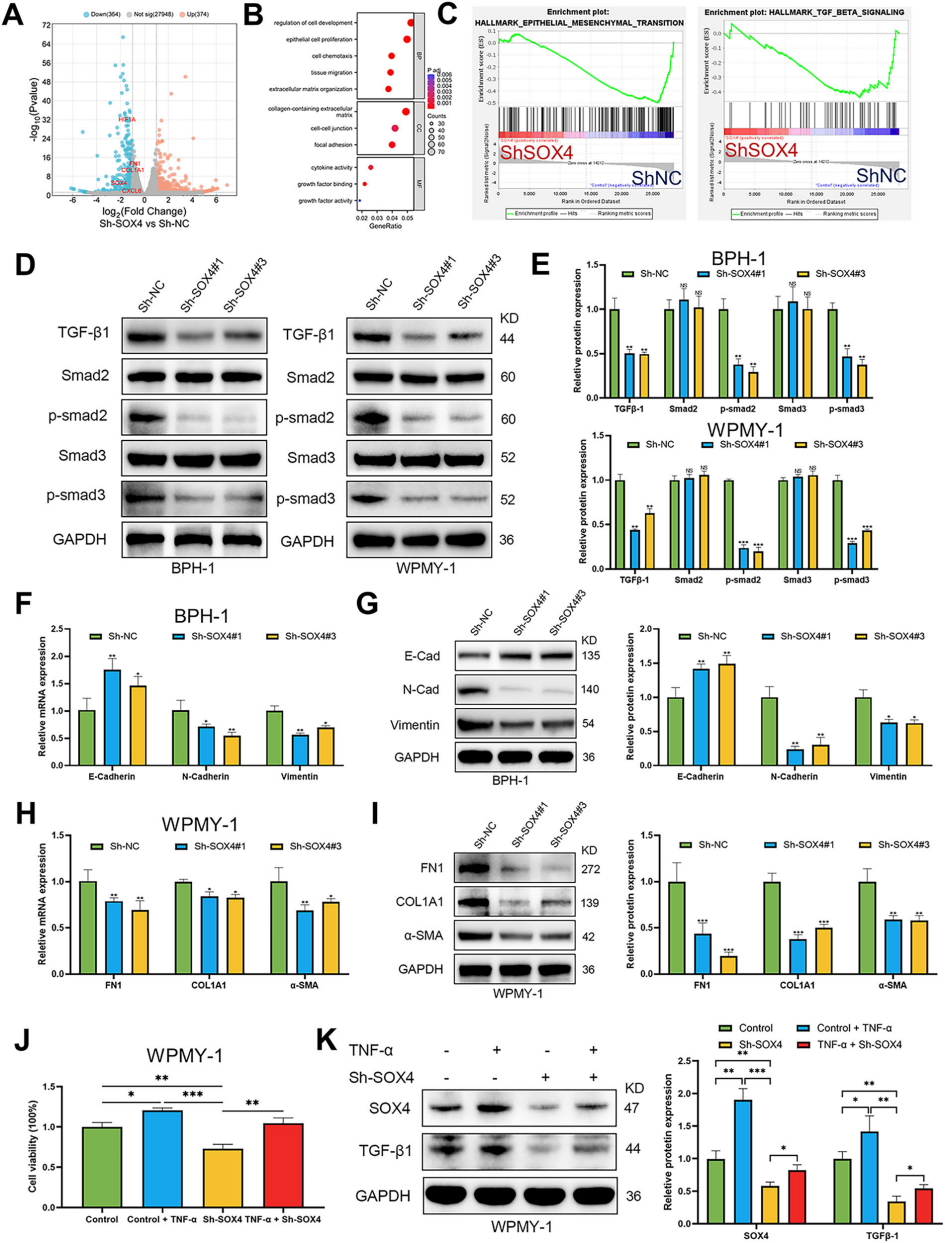
Knockdown of SOX4 regulates TGF-β/Smad signaling pathway and its downstream genes. **A** Valcano plot showing the differentiated expressed genes in WPMY-1 cells knocking down SOX4. **B** GO analysis showing pathways down/up-regulated in WPMY-1 cells knocking down SOX4. **C** Enrichment plots of GSEA analyses for significant pathways in Sh-SOX4 group compared with Sh-NC group in WPMY-1 cells. **D**, **E** WB analysis of TGF-β/Smad pathway protein expression in BPH-1 and WPMY-1 cells infected with Sh-NC or Sh-SOX4 lentivirus for 3 days. **F**, **G** RT-PCR and WB analysis of EMT marker expression in BPH-1 cells infected with Sh-NC or Sh-SOX4 lentivirus for 3 days. **H**, **I** RT-PCR and WB analysis of fibrosis marker expression in WPMY-1 cells infected with Sh-NC or Sh-SOX4 lentivirus for 3 days. **J** WPMY-1 cells were plated in 96-well plates (3 × 10^3^ cells/well) overnight. Then, WPMY-1 cells were pretreated with TNF-α and transfected with SOX4 lentivirus for 3 days. Cells viability was determined by CCK-8 test. **K** WB showing SOX4 and TGF-β1 protein expression in WPMY-1 cells treated with TNF-α and infected with Sh-SOX4 lentivirus for 3 days. Data are expressed as the means ± SEMs (*p < 0.05, **p < 0.01, ***p < 0.001, ns: not significant).

To elucidate the specific mechanism by which SOX4 functions in BPH, we performed RNA-seq analysis on WPMY-1 cells with either silenced or overexpressed SOX4. The results revealed that upon SOX4 knockdown, 364 genes were significantly downregulated and 374 genes were significantly upregulated (Fig. 5A). Similarly, in cells with SOX4 overexpression, 1450 genes were significantly upregulated and 1425 genes were significantly downregulated (Fig. S5A). GO analysis further revealed that SOX4 knockdown primarily affects processes related to the regulation of cell development, epithelial cell proliferation, cell chemotaxis, extracellular matrix organization, focal adhesion, and chemokine activity (Fig. 5B). GSEA enrichment analysis indicated that SOX4 knockdown resulted in the downregulation of pathways associated with EMT and TGF-β signaling compared to the control group (Fig. 5C). Notably, in WPMY-1 cells with SOX4 overexpression, significant changes in extracellular matrix organization, EMT, smooth muscle cell proliferation, positive regulation of inflammatory responses, cell-cell junction, and chemokine activity were observed by GO analysis (Fig. S5B). In addition, GSEA enrichment analysis also showed significant upregulation of pathways such as G2M checkpoint, mTORC1 signaling, androgen response, and E2F targets after overexpression of SOX4 (Fig. S5C). We also found that SOX4 knockdown significantly inhibited the expression of TGF-β1, p-smad2, and p-smad3. The ratios of p-smad2/Smad2 and p-smad3/Smad3 were also decreased (Fig. 5D, E), whereas the opposite results were observed with SOX4 overexpression (Fig. S5D, E). Knockdown of SOX4 in BPH-1 cells also significantly affected the expression of EMT-associated markers, including the upregulation of E-cadherin and the downregulation of N-cadherin and Vimentin (Fig. 5F, G). It was also shown to significantly inhibit the expression of fibrosis markers, such as FN1, COL1A1, and α-SMA in WPMY-1 cells (Fig. 5H, I). Notably, we found that overexpression of SOX4 in BPH-1 cells led to the downregulation of E-cadherin and the upregulation of N-cadherin and Vimentin (Fig. S5F, G). At the same time, it significantly promoted the expression of fibrotic markers such as FN1, COL1A1 and α-SMA in WPMY-1 cells (Fig. S5H, I). To further verify the effect of TNF-α on SOX4, we performed SOX4 knockdown in WPMY-1 cells treated with TNF-α. The CCK-8 assay showed that TNF-α treatment promoted cell proliferation, while knockdown of SOX4 significantly reversed this effect. Additionally, the WB analysis further demonstrated that TNF-α upregulated the expression of SOX4 and TGF-β1, and this effect was significantly reversed by SOX4 knockdown (Fig. 5J, K). Overall, these data suggest that TNF-α can activate the TGF-β1/Smad2/3 signaling pathway by upregulating SOX4, thereby regulating EMT and fibrosis.

### Met inhibits proliferation of BPH cells by suppressing the SOX4/TGF-β/Smad signaling axis

**Fig. 6:**
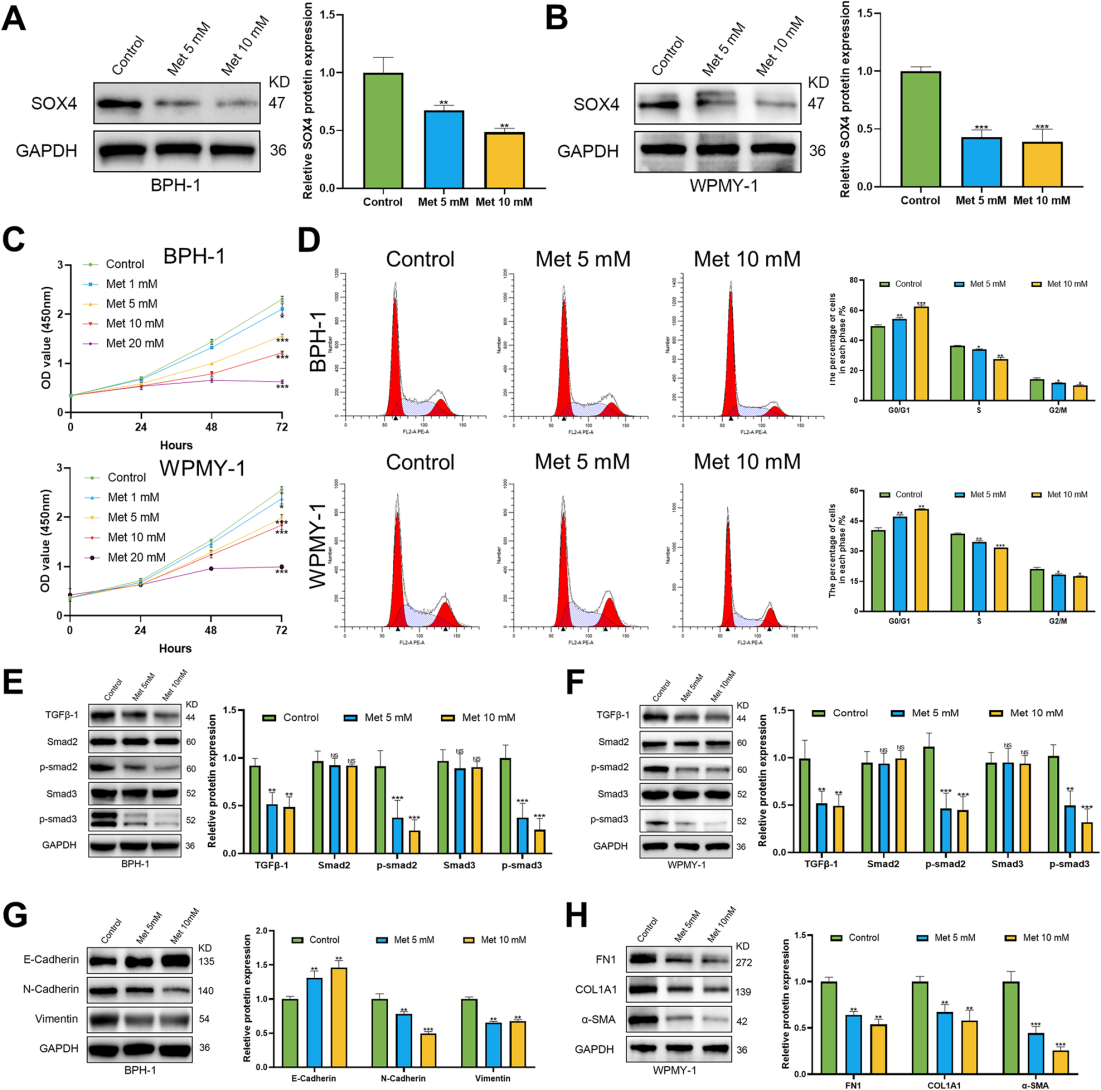
Met inhibits cell viability and cell cycle via the SOX4/TGF-β/Smad signaling axis in BPH-1 and WPMY-1 cells. **A**, **B** WB analysis of SOX4 protein expression in BPH-1 and WPMY-1 cells treated with Met (0, 5, and 10 mM), retrospectively. **C** BPH-1 and WPMY-1 cells were plated in 96-well plates (3 × 10^3^ cells/well) overnight. Then, BPH-1 and WPMY-1 cells were treated with doses of Met (0, 1, 5, 10, and 20 mM) for different time points (0, 24, 48, and 72 h). Cells viability was determined by CCK-8 test. **D** BPH-1 and WPMY-1 cells were plated in 6-well plates (2 × 10^5^ cells/well) overnight. Then, BPH-1 and WPMY-1 cells treated with Met (0, 5, and 10 mM) for 3 days, and harvested for cell cycle test via flow cytometry. **E**, **F** WB analysis of TGF-β/Smad pathway protein expression in BPH-1 and WPMY-1 cells treated with Met (0, 5, and 10 mM) for 3 days. **G** WB analysis of EMT marker expression in BPH-1 cells treated with Met (0, 5, and 10 mM) for 3 days. **H** WB analysis of fibrosis marker expression in WPMY-1 cells treated with Met (0, 5, and 10 mM) for 3 days. Data are expressed as the means ± SEMs (*p < 0.05, **p < 0.01, ***p < 0.001, ns: not significant).

To investigate the function of Met in BPH, we treated BPH-1 and WPMY-1 cells with different doses of Met. The results revealed that Met significantly inhibited the protein expression of SOX4 (Fig. 6A, B). Subsequently, the CCK-8 assay demonstrated that Met significantly inhibited the cell viability of BPH-1 and WPMY-1 cells in a dose-dependent manner, respectively (Fig. 6C, Fig. S6A). Flow cytometry indicated that the proportion of cells in the G0/G1 phase significantly increased after Met treatment, whereas the proportions of cells in the G2/M and S phases decreased (Fig. 6D). Additionally, Met significantly increased the rate of apoptosis (Fig. S6B, C) and led to the downregulation of Bcl2 expression and the upregulation of Bax expression (Fig. S6D, E). Met was shown to significantly inhibit the expression of TGF-β1, p-smad2, and p-smad3, with corresponding decreases in the p-smad2/Smad2 and p-smad3/Smad3 ratios (Fig. 6E, F). We further demonstrated that Met could regulate the expression of EMT-related molecules in BPH-1 cells, including upregulation of E-cadherin and downregulation of N-cadherin and Vimentin (Fig. 6G). Met also significantly reduced the expression of fibrosis markers FN1, COL1A1, and α-SMA in WPMY-1 cells (Fig. 6H). In general, our data indicate that Met could downregulate SOX4 and inhibit the activation of the TGF-β1/Smad2/3 signaling pathway, thereby modulating the EMT process and alleviating fibrosis.

### Overexpression of SOX4 partially reverses the inhibitory effects of Met on BPH cells

**Fig. 7:**
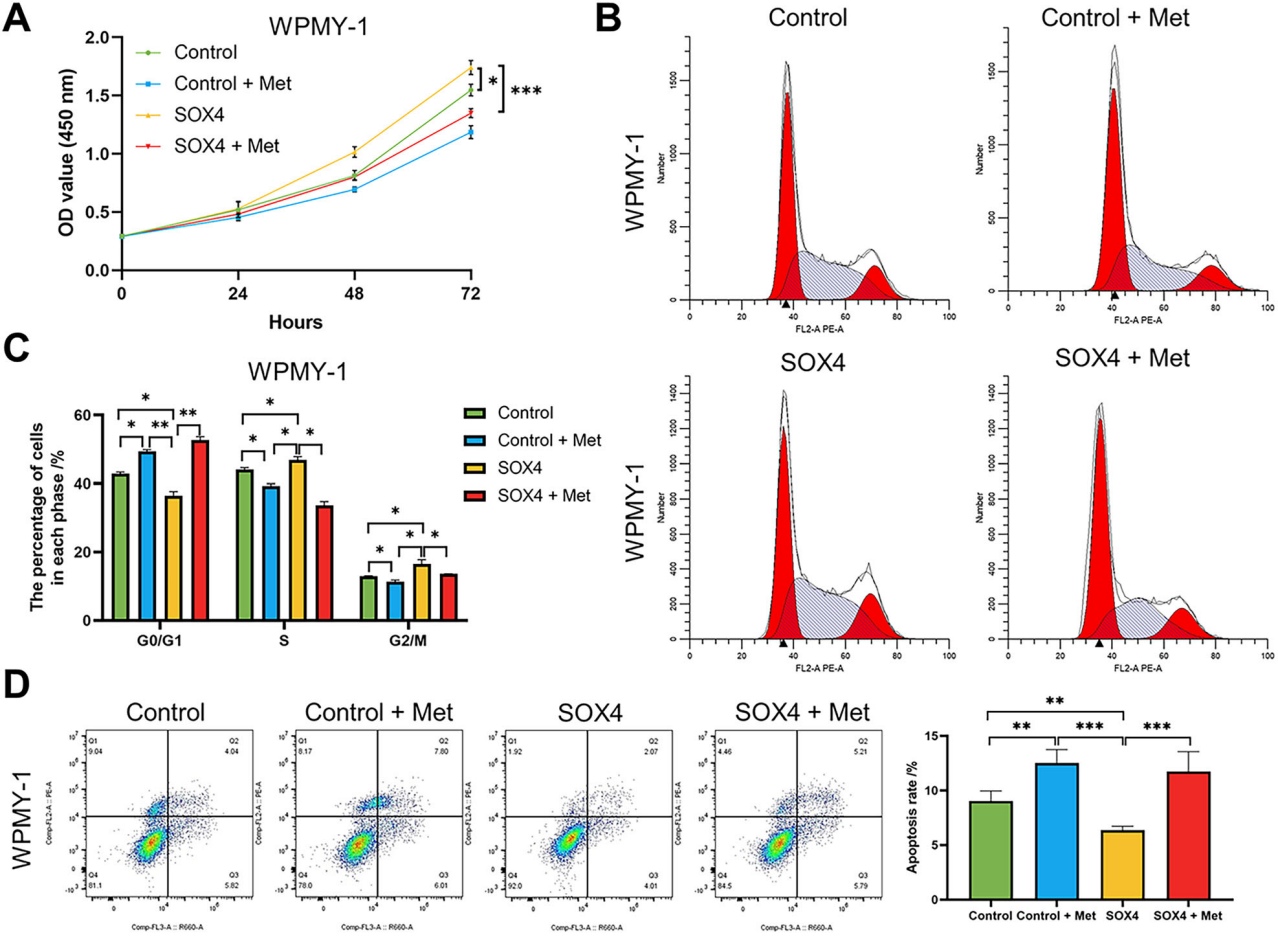
Overexpression of SOX4 partially reverses the effects of Met-induced inhibition of BPH cell proliferation and TGF-β/Smad signaling activation. **A** WPMY-1 cells were plated in 96-well plates (3 × 10^3^ cells/well) overnight. Then, WPMY-1 cells were treated with Vector, Vector combined with Met, overexpression SOX4, or overexpression SOX4 combined with Met. for different time points (0, 24, 48, and 72 h). Cells viability was determined by CCK-8 test. **B**, **C** WPMY-1 cells were plated in 6-well plates (2 × 10^5^ cells/well) overnight. Then, WPMY-1 cells were treated with Vector, Vector combined with Met, overexpression SOX4, or overexpression SOX4 combined with Met for 3 days, and harvested for cell cycle test via flow cytometry. **D** WPMY-1 cells were plated in 6-well plates (2 × 10^5^ cells/well) overnight. Then, WPMY-1 cells were treated with Vector, Vector combined with Met, overexpression SOX4, or overexpression SOX4 combined with Met for 3 days, and harvested for cell apoptosis test via flow cytometry. Data are expressed as the means ± SEMs (*p < 0.05, **p < 0.01, ***p < 0.001).

To further elucidate whether Met effects in BPH by mediating SOX4, we first transfected WPMY-1 cells with either SOX4 overexpression or empty plasmids, and then treated the cells with Met. The results demonstrated that SOX4 overexpression significantly promoted WPMY-1 cell proliferation, while the combination of Met treatment and SOX4 overexpression inhibited the cell viability (Fig. 7A). Moreover, SOX4 overexpression facilitated the transition of G0/G1 phase, whereas the combination of Met treatment and SOX4 overexpression had the opposite effect (Fig. 7B, C). SOX4 overexpression also reduced the rate of apoptosis, while cotreatment with Met significantly promoted apoptosis (Fig. 7D). Furthermore, SOX4 overexpression promoted the expression of fibrosis markers (COL1A1 and α-SMA) in WPMY-1 cells, and this effect was notably reversed by Met treatment (Fig. S7A). The study also found that SOX4 overexpression significantly upregulated the expression of TGF-β1, p-smad2, and p-smad3, which was counteracted by Met (Fig. S7B). These findings suggest that Met inhibits prostate cell proliferation by downregulating SOX4 and subsequently inhibiting the TGF-β1/Smad2/3 signaling axis.

### Met demonstrates therapeutic effects for BPH via inhibiting the SOX4/TGF-β/Smad signaling axis in vivo

**Fig. 8:**
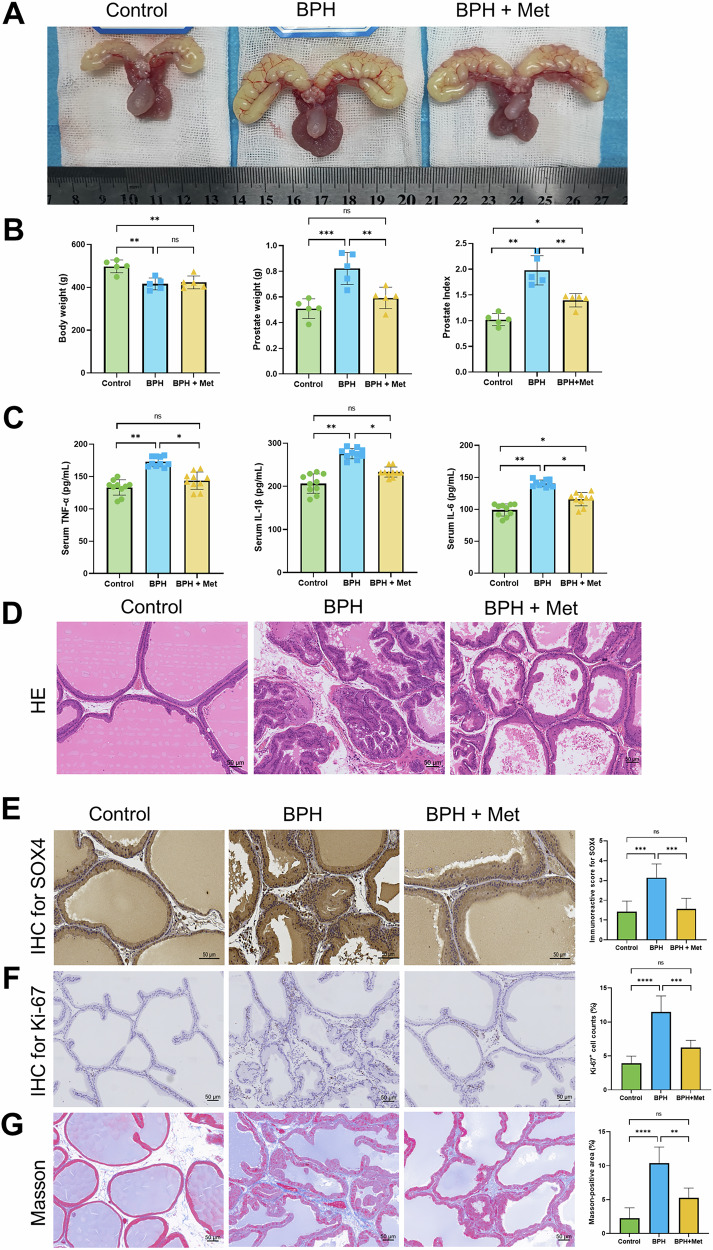
Met exerts its inhibitory effects by modulating the SOX4/TGF-β/Smad signaling axis in vivo. **A** Prostate pictures in control, BPH, and BPH combined with Met groups, retrospectively. **B** Representative HE staining of prostate samples in control, BPH, and BPH combined with Met group. **C** Bar plots showing the body weight, prostate weight, and prostate index in control, BPH, and BPH combined with Met groups. **D** Bar plots showing the serum levels of TNF-α, IL-1β, and IL-6 in control, BPH, and BPH combined with Met groups. **E** Representative SOX4 IHC staining of prostate samples in control, BPH, and BPH combined with Met groups. **F** Representative IHC staining of Ki-67 in prostate samples from the control, BPH, and BPH combined with Met groups. **G** Representative Masson staining of prostate samples from the control, BPH, and BPH combined with Met groups. Data are expressed as the means ± SEMs (*p < 0.05, **p < 0.01, ***p < 0.001, ****p < 0.0001, ns not significant).

As described above, we established a TP-induced BPH rat model and investigated the in vivo effects of Met on BPH through oral administration (Figs. 8A and S8A). Compared with the control rats, TP-induced BPH rats presented significant increases in prostate weight (PW) and prostate index (PI), while Met treatment significantly reduced the PW and PI in these rats (Fig. 8B). We also observed that the serum levels of proinflammatory cytokines TNF-α, IL-1β, and IL-6 in the BPH group were significantly higher than those in the control group (Fig. 8C), and there was a positive correlation between TNF-α concentration and the expression of IL-1β and IL-6 (Fig. S8B). In addition, treatment with Met significantly decreased the expression of these proinflammatory cytokines (Fig. 8C). Regarding the pathological features of prostate tissues, compared to the control group, prostate tissue sections from BPH rats presented an increased number of acini, a reduced lumen size, and a significant increase in the thickness of both the epithelial layer surrounding the glands and the stromal components (Fig. 8D). Further IHC staining revealed that SOX4 expression was significantly elevated in the BPH group, but markedly reduced after Met treatment (Fig. 8E). Similarly, the proliferation marker Ki-67 was significantly upregulated in the BPH group, while Met decreased its expression (Fig. 8F). Additionally, Masson staining revealed an increased fibrosis ratio in the prostate tissues of the BPH rats compared to the control rats, with Met significantly reducing the fibrosis ratio (Fig. 8G). We also assessed the expression of TGF-β1, EMT, and fibrosis markers in the prostate tissues of the rats. As illustrated, TGF-β1 expression was significantly greater in BPH group compared to the control group, and Met resulted in a notable reduction in TGF-β1 expression (Fig. S8C). Similarly, Met significantly reduced the expression of FN1 and α-SMA in BPH rats (Fig. S8D). In terms of EMT related proteins, BPH rats exhibited decreased E-cadherin and increased N-cadherin and Vimentin levels. Met treatment reversed these changes, leading to increased E-cadherin and decreased N-cadherin and Vimentin levels (Fig. S8E). The results further revealed that protein levels of TGF-β1, p-smad2, and p-smad3 were significantly elevated in BPH rats, but were reversed by Met (Fig. S8F). It is important to highlight that the BPH group exhibited elevated levels of SOX4 and fibrosis markers, including FN1, COL1A1, and α-SMA (Fig. S8G), which were significantly reduced after Met treatment. Additionally, the BPH group showed an increase in N-cadherin and Vimentin expression, along with a decrease in E-Cadherin. In contrast, the BPH + Met group displayed a reduction in N-cadherin and Vimentin, accompanied by an increase in E-Cadherin (Fig. S8H). Taken together, these data confirm that Met may exert its potential anti-BPH effects by modulating the TGF-β1/Smad2/3 pathway through the inhibition of SOX4, thereby reversing prostate EMT and alleviating fibrosis.

## Discussion

Increasing research indicates that immune-inflammatory cells and their secreted proinflammatory cytokines promote pathological changes in the prostate by inducing tissue remodeling, fibrosis, and cellular proliferation [33]. Chronic inflammation not only exacerbates LUTS but also correlates with a higher risk of AUR in BPH patients [13, 34]. Moreover, severe prostatic inflammation has been shown to reduce the therapeutic efficacy of 5α-reductase inhibitors (5ARIs) or α-adrenergic antagonists (ARs) in treating BPH [34]. Therefore, inflammation is considered a key driver of BPH progression, although its precise mechanisms remain unclear. As a major mediator of inflammatory responses, TNF-α plays a crucial role in homeostasis and disease pathogenesis [35]. Existing evidence indicates that TNF-α can induce inflammation, activate the vascular endothelium, coordinate the recruitment of immune cells, and promote tissue destruction [36]. Persistent inflammatory damage and chronic tissue healing are significant factors contributing to BPH development [37]. Therefore, TNF-α-mediated inflammatory mechanisms may play essential roles in BPH.

In this study, we found that TNF-α is highly expressed in BPH patients and significantly promotes prostate cell proliferation. The results also revealed that TNF-α enhances the expression of the inflammatory cytokines IL-1α, IL-1β, IL-6, IL-8, and IL-18 in cells. These cytokines are considered bridges between inflammation and hyperplasia, as they intersect with key paracrine signaling pathways that regulate prostate growth. IL-8 is a paracrine inducer of FGF-2, which has been shown to be an effective growth factor for stromal and epithelial cells in BPH [38]. Additionally, IL-1 and IL-6 can induce the JAK-STAT signaling pathway, thereby driving various physiological and pathological processes in the prostate, including proliferation, the immune response, and inflammation [39–41].

Previous studies have shown that under inflammatory stimulation, cells undergo a series of morphological and biochemical changes, during which they lose epithelial characteristics and transdifferentiate into mesenchymal cells [42–44]. Therefore, inflammation is considered an effective inducer of EMT. Palafox-Mariscal et al. [42] reported TNF-α induces EMT in cervical cancer cells, and anti-inflammatory compounds may be an option to disrupt EMT. Adachi et al. [43] demonstrated TNF-α can induce EMT in renal tubular epithelial cells, potentially exacerbating renal interstitial fibrosis in glomerulonephritis. Yoshimatsu et al. [44] discovered TNF-α enhances TGF-β-induced EMT in human endothelial cells through the TGF-β signaling pathway. Furthermore, fibrosis is considered the final common pathophysiological change in chronic inflammatory diseases, where various proinflammatory signals can induce the EMT process, activating a pathological fibrotic state [45, 46]. Studies have indicated that long-term activation of the EMT process in response to injury can provoke inflammation and lead to severe fibrosis, ultimately resulting in delayed wound healing of epithelial tissues and destruction of organ tissue structure. The EMT process has been reported to be associated with fibrotic diseases in multiple organs, such as the lungs, kidneys, and liver [47–49]. Notably, recent studies have reported that inflammation-induced tissue stress increases the activation of the RhoA/ROCK1/F-actin/YAP1 axis, thereby promoting prostate cell survival and fibrosis, which accelerates BPH progression [50]. Our data also strongly support TNF-α enhances the EMT process in epithelial cells and fibrosis in stromal cells.

SOX4 is a multifunctional transcription factor that plays a crucial role in cell fate determination, proliferation, and differentiation. It has been reported that SOX4 is frequently amplified and overexpressed in various malignancies [51], strongly supporting the notion that SOX4 is an oncogene, including in breast, bladder, and prostate cancers [24, 52, 53]. These findings suggest that the tumorigenic role of SOX4 is attributed primarily to the inhibition of apoptosis, promotion of cell survival, direct coordination of mesenchymal marker induction, and indirect regulation and activation by other EMT inducers [54, 55]. However, its role in BPH has not been fully determined. The balance between cell proliferation and apoptosis is fundamental to growth homeostasis, and any disruption of this balance may lead to the progression of BPH [56]. Our results indicated that TNF-α significantly promoted the expression of SOX4, and SOX4 was highly expressed in hyperplastic prostate tissue. Further experiments revealed that knocking down SOX4 significantly inhibited the proliferative activity of prostate cells and led to a substantial increase in the apoptosis ratio. Additionally, silencing SOX4 induced cell cycle arrest, which was accompanied by a significant downregulation of cell cycle-related proteins.

We further explored the potential mechanisms by which TNF-α regulates prostate cell proliferation, EMT, and fibrosis. Current evidence indicates that the TGF-β signaling pathway plays a crucial role in cell proliferation, differentiation, and tissue remodeling [57]. TGF-β1, the primary ligand of this pathway, binds to its receptors to activate downstream SMAD proteins, thereby regulating gene expression and cellular behavior [58]. Additionally, the TGF-β signaling pathway has been shown to inhibit epithelial characteristics and enhance mesenchymal features through the transcriptional regulation of transcription factors, making it a major inducer of EMT [55]. Previous studies have demonstrated that TGF-β1 promotes BPH progression by regulating the EMT process in the prostate epithelium [59, 60]. Recent studies have also revealed that TGF-β1, a key regulator of fibrosis, promotes fibroblast activation and ECM deposition, thereby exacerbating prostate fibrosis [61, 62]. Thus, TGF-β1 may play an important role in the development and progression of BPH. Interestingly, SOX4, as a core component of TGF-β signal transduction, mediates EMT in various types of cancers [63]. In the present study, we confirmed that TNF-α upregulates SOX4 expression, which further activates the TGF-β1/Smad2/3 axis, leading to proliferation, EMT processes, and fibrosis in prostate cells. Additionally, SOX4 knockdown significantly inhibited EMT processes, fibrosis, and activation of the TGF-β1/Smad2/3 signaling pathway, whereas SOX4 overexpression reversed these effects. This mechanism reveals that TNF-αnot only acts as a proinflammatory factor in BPH but also directly promotes pathological changes in prostate tissue by regulating key transcription factors and signaling pathways. At the same time, these findings strongly suggest that SOX4 could be a potential therapeutic target for treating BPH.

Although current guidelines recommend ARs, phosphodiesterase 5 inhibitors, and 5ARIs for the treatment of BPH [64, 65], the efficacy of these medications remains unsatisfactory, leaving significant room for improvement in the treatment outcomes and quality of life for BPH patients. In recent years, the mechanisms of action of Met, a basic hypoglycemic drug, have been widely reported, and its antitumor effects as a potential treatment for certain cancers have been explored in the field of oncology. These studies indicate Met inhibits tumor growth by suppressing mitochondrial OXPHOS through both AMPK-dependent and AMPK-independent mechanisms [66, 67]. Moreover, substantial evidence shows that Met can exert its effects by inhibiting the expression of SOX4, thereby inhibiting the progression of diseases such as breast cancer, lung cancer, and atherosclerosis [30, 68, 69]. Of note, Met has also been found to potentially alleviate BPH. The study demonstrated that oral administration of 500 or 1000 mg/kg Met for 14 days significantly inhibited testosterone-mediated increases in PW and PI and alleviated testosterone-induced pathological changes [31]. Wang et al. [70] found that Met inhibits the proliferation of prostate epithelial cells by suppressing the expression of insulin-like growth factor 1 (IGF-1) and IGF-1R in cells. However, the mechanism by which Met regulates BPH remains unclear. This study revealed that Met can significantly downregulate SOX4 expression and inhibit the activation of the TGF-β1/Smad2/3 signaling pathway, thereby inhibiting prostate cell proliferation, EMT transformation, and fibrosis. We then treated SOX4-overexpressing WPMY-1 cells with Met, and the results indicated that the activation effect of SOX4 overexpression was completely reversed by Met. In a rat model, we further confirmed that Met alleviated testosterone-induced pathological changes in BPH tissue, inhibited SOX4 expression, and reduced cell proliferation, EMT process, and fibrosis. Although there are currently no specific inhibitors targeting SOX4, new data suggest that Met can indirectly target and inhibit SOX4 expression, leading to the suppression of its downstream TGF-β1/Smad2/3 signaling pathway. Therefore, Met has the potential to target and treat BPH by inhibiting SOX4.

Although this study analyzed the potential mechanism by which TNF-α influences the progression of BPH through the upregulation of SOX4, several limitations remain. First, this study primarily explored the TNF-α-induced inflammation model and did not fully consider the potential roles of other proinflammatory factors in BPH progression. Second, although the RNA-seq results of this study indicated that TNF-α upregulates the expression of SOX4, we have not been able to demonstrated that TNF-α can directly regulate SOX4. Moreover, further experiments are needed to determine whether SOX4 transcriptionally activates inflammatory factors, thereby promoting the formation and maintenance of an inflammatory microenvironment, and driving prostate cell proliferation and tissue remodeling.

Collectively, this study demonstrated the critical role of TNF-α promoting prostate cell proliferation and fibrosis via upregulating SOX4/TGF-β/Smad signaling axis. Additionally, the findings also indicated that Met is a potential inhibitor of SOX4 and a promising therapeutic agent for BPH treatment. Our research further elucidates the inflammatory regulatory mechanisms of BPH and provides new insights and targets for the pathogenesis and clinical treatment of BPH.

## Supplementary information

Supplementary Information

Original images of western blot gel

## Data Availability

The datasets used and/or analyzed during the current study are available from the corresponding author on reasonable request.

## References

[1] Devlin CM, Simms MS, Maitland NJ. Benign prostatic hyperplasia - what do we know? BJU Int. 2021;127:389–99. 32893964 10.1111/bju.15229

[2] Hoke GP, McWilliams GW. Epidemiology of benign prostatic hyperplasia and comorbidities in racial and ethnic minority populations. Am J Med. 2008;121:S3–10. 18675615 10.1016/j.amjmed.2008.05.021

[3] Vuichoud C, Loughlin KR. Benign prostatic hyperplasia: epidemiology, economics and evaluation. Can J Urol. 2015;22:1–6. 26497338

[4] DerSarkissian M, Xiao Y, Duh MS, Lefebvre P, Swensen AR, Bell CF. Comparing clinical and economic outcomes associated with early initiation of combination therapy of an alpha blocker and dutasteride or finasteride in men with benign prostatic hyperplasia in the United States. J Manag Care Spec Pharm. 2016;22:1204–14. 27668569 10.18553/jmcp.2016.22.10.1204 PMC10397950

[5] Chang WH, Tsai YS, Wang JY, Chen HL, Yang WH, Lee CC. Sex hormones and oxidative stress mediated phthalate-induced effects in prostatic enlargement. Environ Int. 2019;126:184–92. 30798199 10.1016/j.envint.2019.02.006

[6] Untergasser G, Madersbacher S, Berger P. Benign prostatic hyperplasia: age-related tissue-remodeling. Exp Gerontol. 2005;40:121–8. 15763388 10.1016/j.exger.2004.12.008

[7] Vignozzi L, Rastrelli G, Corona G, Gacci M, Forti G, Maggi M. Benign prostatic hyperplasia: a new metabolic disease?. J Endocrinol Investig. 2014;37:313–22. 24458832 10.1007/s40618-014-0051-3

[8] Liedtke V, Stöckle M, Junker K, Roggenbuck D. Benign prostatic hyperplasia - a novel autoimmune disease with a potential therapy consequence?. Autoimmun Rev. 2024;23:103511. 38168573 10.1016/j.autrev.2023.103511

[9] Robert G, Descazeaud A, Nicolaïew N, Terry S, Sirab N, Vacherot F, et al. Inflammation in benign prostatic hyperplasia: a 282 patients’ immunohistochemical analysis. Prostate. 2009;69:1774–80. 19670242 10.1002/pros.21027 PMC2833181

[10] Manzarbeitia F, Vela Navarrete R, Fernandez-Acenero MJ. Early histopathological aspects of benign prostatic hyperplasia: myxoid-inflammatory nodules]. Actas Urol Esp. 2010;34:549–54. 20510119

[11] Fibbi B, Penna G, Morelli A, Adorini L, Maggi M. Chronic inflammation in the pathogenesis of benign prostatic hyperplasia. Int J Androl. 2010;33:475–88. 19508330 10.1111/j.1365-2605.2009.00972.x

[12] Fan Y, Yang L, Wei Q, Ding Y, Tang Z, Tan P, et al. Toll-like receptor 10 (TLR10) exhibits suppressive effects on inflammation of prostate epithelial cells. Asian J Androl. 2019;21:393–9. 30618413 10.4103/aja.aja_100_18 PMC6628737

[13] Li J, Li Y, Cao D, Huang Y, Peng L, Meng C, et al. The association between histological prostatitis and benign prostatic hyperplasia: a single-center retrospective study. Aging Male. 2022;25:88–93. 35289705 10.1080/13685538.2022.2050360

[14] Gandaglia G, Briganti A, Gontero P, Mondaini N, Novara G, Salonia A, et al. The role of chronic prostatic inflammation in the pathogenesis and progression of benign prostatic hyperplasia (BPH). BJU Int. 2013;112:432–41. 23650937 10.1111/bju.12118

[15] Naiyila X, Li J, Huang Y, Chen B, Zhu M, Li J, et al. A novel insight into the immune-related interaction of inflammatory cytokines in benign prostatic hyperplasia. J Clin Med. 2023;12:1821. 36902608 10.3390/jcm12051821 PMC10003138

[16] Zelová H, Hošek J. TNF-α signalling and inflammation: interactions between old acquaintances. Inflamm Res. 2013;62:641–51. 23685857 10.1007/s00011-013-0633-0

[17] Sedger LM, McDermott MF. TNF and TNF-receptors: From mediators of cell death and inflammation to therapeutic giants - past, present and future. Cytokine Growth Factor Rev. 2014;25:453–72. 25169849 10.1016/j.cytogfr.2014.07.016

[18] Tong Y, Guo YJ, Zhang Q, Bi HX, Kai K, Zhou RY. Combined treatment with dihydrotestosterone and lipopolysaccharide modulates prostate homeostasis by upregulating TNF-α from M1 macrophages and promotes proliferation of prostate stromal cells. Asian J Androl. 2022;24:513–20. 34975070 10.4103/aja2021114 PMC9491040

[19] Vickman RE, Aaron-Brooks L, Zhang R, Lanman NA, Lapin B, Gil V, et al. TNF is a potential therapeutic target to suppress prostatic inflammation and hyperplasia in autoimmune disease. Nature Commun. 2022;13:2133. 35440548 10.1038/s41467-022-29719-1 PMC9018703

[20] Sinner D, Kordich JJ, Spence JR, Opoka R, Rankin S, Lin SC, et al. Sox17 and Sox4 differentially regulate beta-catenin/T-cell factor activity and proliferation of colon carcinoma cells. Mol Cell Biol. 2007;27:7802–15. 17875931 10.1128/MCB.02179-06 PMC2169141

[21] Moreno CS. SOX4: the unappreciated oncogene. Semin Cancer Biol. 2020;67:57–64. 31445218 10.1016/j.semcancer.2019.08.027 PMC7043201

[22] Andersen CL, Christensen LL, Thorsen K, Schepeler T, Sørensen FB, Verspaget HW, et al. Dysregulation of the transcription factors SOX4, CBFB and SMARCC1 correlates with outcome of colorectal cancer. Br J Cancer. 2009;100:511–23. 19156145 10.1038/sj.bjc.6604884 PMC2658541

[23] Zhang J, Xiao C, Feng Z, Gong Y, Sun B, Li Z, et al. SOX4 promotes the growth and metastasis of breast cancer. Cancer Cell Int. 2020;20:468. 33005101 10.1186/s12935-020-01568-2 PMC7523060

[24] Wang L, Zhang J, Yang X, Chang YW, Qi M, Zhou Z, et al. SOX4 is associated with poor prognosis in prostate cancer and promotes epithelial-mesenchymal transition in vitro. Prostate Cancer Prostatic Dis. 2013;16:301–7. 23917306 10.1038/pcan.2013.25

[25] Ye X, Yin C, Huang X, Huang Y, Ding L, Jin M, et al. ROS/TGF-β signal mediated accumulation of SOX4 in OA-FLS promotes cell senescence. Exp Gerontol. 2021;156:111616. 34742854 10.1016/j.exger.2021.111616

[26] Bhattaram P, Muschler G, Wixler V, Lefebvre V. Inflammatory cytokines stabilize SOXC transcription factors to mediate the transformation of fibroblast-like synoviocytes in arthritic disease. Arthritis Rheumatol. 2018;70:371–82. 29193895 10.1002/art.40386 PMC5826855

[27] Jia M, Li Q, Guo J, Shi W, Zhu L, Huang Y, et al. Deletion of BACH1 attenuates atherosclerosis by reducing endothelial inflammation. Circ Res. 2022;130:1038–55. 35196865 10.1161/CIRCRESAHA.121.319540

[28] Cufí S, Vazquez-Martin A, Oliveras-Ferraros C, Martin-Castillo B, Joven J, Menendez JA. Metformin against TGFβ-induced epithelial-to-mesenchymal transition (EMT): from cancer stem cells to aging-associated fibrosis. Cell Cycle. 2010;9:4461–8. 21088486 10.4161/cc.9.22.14048

[29] Feng YY, Wang Z, Pang H. Role of metformin in inflammation. Mol Biol Rep. 2023;50:789–98. 36319785 10.1007/s11033-022-07954-5

[30] Cheng CK, Lin X, Pu Y, Tse JKY, Wang Y, Zhang CL, et al. SOX4 is a novel phenotypic regulator of endothelial cells in atherosclerosis revealed by single-cell analysis. J Adv Res. 2023;43:187–203. 36585108 10.1016/j.jare.2022.02.017 PMC9811326

[31] Mosli HH, Esmat A, Atawia RT, Shoieb SM, Mosli HA, Abdel-Naim AB. Metformin attenuates testosterone-induced prostatic hyperplasia in rats: a pharmacological perspective. Sci Rep. 2015;5:15639. 26492952 10.1038/srep15639 PMC4616049

[32] Varghese F, Bukhari AB, Malhotra R, De A. IHC Profiler: an open source plugin for the quantitative evaluation and automated scoring of immunohistochemistry images of human tissue samples. PLoS ONE. 2014;9:e96801. 24802416 10.1371/journal.pone.0096801 PMC4011881

[33] Devlin CM, Simms MS, Maitland NJ. Benign prostatic hyperplasia – what do we know?. BJU Int. 2021;127:389–99. 32893964 10.1111/bju.15229

[34] Torkko KC, Wilson RS, Smith EE, Kusek JW, van Bokhoven A, Lucia MS. Prostate biopsy markers of inflammation are associated with risk of clinical progression of benign prostatic hyperplasia: findings from the MTOPS study. J Urol. 2015;194:454–61. 25828974 10.1016/j.juro.2015.03.103

[35] Bradley JR. TNF-mediated inflammatory disease. J Pathol. 2008;214:149–60. 18161752 10.1002/path.2287

[36] Feldmann M. Translating molecular insights in autoimmunity into effective therapy. Annu Rev Immunol. 2009;27:1–27. 19007330 10.1146/annurev-immunol-082708-100732

[37] De Nunzio C, Kramer G, Marberger M, Montironi R, Nelson W, Schröder F, et al. The controversial relationship between benign prostatic hyperplasia and prostate cancer: the role of inflammation. Eur Urol. 2011;60:106–17. 21497433 10.1016/j.eururo.2011.03.055

[38] Giri D, Ittmann M. Interleukin-8 is a paracrine inducer of fibroblast growth factor 2, a stromal and epithelial growth factor in benign prostatic hyperplasia. Am J Pathol. 2001;159:139–47. 11438462 10.1016/S0002-9440(10)61681-1 PMC1850405

[39] Jerde TJ, Bushman W. IL-1 induces IGF-dependent epithelial proliferation in prostate development and reactive hyperplasia. Sci Signal. 2009;2:ra49. 19724062 10.1126/scisignal.2000338 PMC2949294

[40] Hahn AM, Myers JD, McFarland EK, Lee S, Jerde TJ. Interleukin-driven insulin-like growth factor promotes prostatic inflammatory hyperplasia. J Pharm Exp Ther. 2014;351:605–15. 10.1124/jpet.114.218693 PMC4244580 25292180

[41] Shankar E, Bhaskaran N, MacLennan GT, Liu G, Daneshgari F, Gupta S. Inflammatory signaling involved in high-fat diet induced prostate diseases. J Urol Res. 2015;2:1010. PMC4583131 26417612

[42] Palafox-Mariscal LA, Ortiz-Lazareno PC, Jave-Suárez LF, Aguilar-Lemarroy A, Villaseñor-García MM, Cruz-Lozano JR, et al. Pentoxifylline inhibits TNF-α/TGF-β1-induced epithelial-mesenchymal transition via suppressing the NF-κB pathway and SERPINE1 expression in CaSki cells. Int J Mol Sci. 2023;24:10592. 37445768 10.3390/ijms241310592 PMC10342099

[43] Adachi T, Arito M, Suematsu N, Kamijo-Ikemori A, Omoteyama K, Sato T, et al. Roles of layilin in TNF-α-induced epithelial-mesenchymal transformation of renal tubular epithelial cells. Biochem Biophys Res Commun. 2015;467:63–69. 26410531 10.1016/j.bbrc.2015.09.121

[44] Yoshimatsu Y, Wakabayashi I, Kimuro S, Takahashi N, Takahashi K, Kobayashi M, et al. TNF-α enhances TGF-β-induced endothelial-to-mesenchymal transition via TGF-β signal augmentation. Cancer Sci. 2020;111:2385–99. 32385953 10.1111/cas.14455 PMC7385392

[45] Thiery JP, Acloque H, Huang RY, Nieto MA. Epithelial-mesenchymal transitions in development and disease. Cell. 2009;139:871–90. 19945376 10.1016/j.cell.2009.11.007

[46] Wynn TA, Ramalingam TR. Mechanisms of fibrosis: therapeutic translation for fibrotic disease. Nat Med. 2012;18:1028–40. 22772564 10.1038/nm.2807 PMC3405917

[47] Chilosi M, Poletti V, Zamò A, Lestani M, Montagna L, Piccoli P, et al. Aberrant Wnt/beta-catenin pathway activation in idiopathic pulmonary fibrosis. Am J Pathol. 2003;162:1495–502. 12707032 10.1016/s0002-9440(10)64282-4 PMC1851206

[48] Zeisberg M, Yang C, Martino M, Duncan MB, Rieder F, Tanjore H, et al. Fibroblasts derive from hepatocytes in liver fibrosis via epithelial to mesenchymal transition. J Biol Chem. 2007;282:23337–47. 17562716 10.1074/jbc.M700194200

[49] Zeisberg M, Hanai J, Sugimoto H, Mammoto T, Charytan D, Strutz F, et al. BMP-7 counteracts TGF-beta1-induced epithelial-to-mesenchymal transition and reverses chronic renal injury. Nat Med. 2003;9:964–8. 12808448 10.1038/nm888

[50] Lin D, Luo C, Wei P, Zhang A, Zhang M, Wu X, et al. YAP1 recognizes inflammatory and mechanical cues to exacerbate benign prostatic hyperplasia via promoting cell survival and fibrosis. Adv Sci. 2024;11:e2304274. 10.1002/advs.202304274 PMC10837380 38050650

[51] Rhodes DR, Yu J, Shanker K, Deshpande N, Varambally R, Ghosh D, et al. Large-scale meta-analysis of cancer microarray data identifies common transcriptional profiles of neoplastic transformation and progression. Proc Natl Acad Sci USA. 2004;101:9309–14. 15184677 10.1073/pnas.0401994101 PMC438973

[52] Yu K, Ganesan K, Tan LK, Laban M, Wu J, Zhao XD, et al. A precisely regulated gene expression cassette potently modulates metastasis and survival in multiple solid cancers. PLoS Genet. 2008;4:e1000129. 18636107 10.1371/journal.pgen.1000129 PMC2444049

[53] Weinstein JN, Collisson EA, Mills GB, Shaw KR, Ozenberger BA, Ellrott K, et al. The Cancer Genome Atlas Pan-Cancer analysis project. Nat Genet. 2013;45:1113–20. 24071849 10.1038/ng.2764 PMC3919969

[54] Wu X, Xin Z, Zou Z, Lu C, Yu Z, Feng S, et al. SRY-related high-mobility-group box 4: Crucial regulators of the EMT in cancer. Semin Cancer Biol. 2020;67:114–21. 31199986 10.1016/j.semcancer.2019.06.008

[55] Lourenço AR, Coffer PJ. SOX4: joining the master regulators of epithelial-to-mesenchymal transition?. Trends Cancer. 2017;3:571–82. 28780934 10.1016/j.trecan.2017.06.002

[56] Liu J, Liu D, Zhang X, Li Y, Fu X, He W, et al. NELL2 modulates cell proliferation and apoptosis via ERK pathway in the development of benign prostatic hyperplasia. Clin Sci. 2021;135:1591–608. 10.1042/CS20210476 34195782

[57] Massagué J. TGFbeta in cancer. Cell. 2008;134:215–30. 18662538 10.1016/j.cell.2008.07.001 PMC3512574

[58] Ikushima H, Miyazono K. TGFbeta signalling: a complex web in cancer progression. Nat Rev Cancer. 2010;10:415–24. 20495575 10.1038/nrc2853

[59] Alonso-Magdalena P, Brössner C, Reiner A, Cheng G, Sugiyama N, Warner M, et al. A role for epithelial-mesenchymal transition in the etiology of benign prostatic hyperplasia. Proc Natl Acad Sci USA. 2009;106:2859–63. 19196965 10.1073/pnas.0812666106 PMC2650376

[60] Hu S, Yu W, Lv TJ, Chang CS, Li X, Jin J. Evidence of TGF-β1 mediated epithelial-mesenchymal transition in immortalized benign prostatic hyperplasia cells. Mol Membr Biol. 2014;31:103–10. 24650126 10.3109/09687688.2014.894211

[61] Khan A, Alzahrani HA, Felemban SG, Algarni AS, Alenezi ABS, Kamal M, et al. Exploring TGF-β signaling in benign prostatic hyperplasia: from cellular senescence to fibrosis and therapeutic implications. Biogerontology. 2025;26:79. 40159577 10.1007/s10522-025-10226-x

[62] Li Y, Li J, Zhou L, Wang Z, Jin L, Cao J, et al. Aberrant activation of TGF-β/ROCK1 enhances stemness during prostatic stromal hyperplasia. Cell Commun Signal. 2024;22:257. 38711089 10.1186/s12964-024-01644-4 PMC11071275

[63] Heldin CH, Vanlandewijck M, Moustakas A. Regulation of EMT by TGFβ in cancer. FEBS Lett. 2012;586:1959–70. 22710176 10.1016/j.febslet.2012.02.037

[64] Gratzke C, Bachmann A, Descazeaud A, Drake MJ, Madersbacher S, Mamoulakis C, et al. EAU Guidelines on the assessment of non-neurogenic male lower urinary tract symptoms including benign prostatic obstruction. Eur Urol. 2015;67:1099–109. 25613154 10.1016/j.eururo.2014.12.038

[65] Sandhu JS, Bixler BR, Dahm P, Goueli R, Kirkby E, Stoffel JT, et al. Management of lower urinary tract symptoms attributed to benign prostatic hyperplasia (BPH): AUA guideline amendment 2023. J Urol. 2024;211:11–19. 37706750 10.1097/JU.0000000000003698

[66] Misirkic Marjanovic MS, Vucicevic LM, Despotovic AR, Stamenkovic MM, Janjetovic KD. Dual anticancer role of metformin: an old drug regulating AMPK dependent/independent pathways in metabolic, oncogenic/tumorsuppresing and immunity context. Am J Cancer Res. 2021;11:5625–43. 34873484 PMC8640802

[67] Wu Z, Wang W, Wei L, Zhu S. Current status and frontier tracking of clinical trials on Metformin for cancer treatment. J Cancer Res Clin Oncol. 2023;149:16931–46. 37698682 10.1007/s00432-023-05391-w PMC11798064

[68] Shi P, Liu W, Tala, Wang H, Li F, Zhang H, et al. Metformin suppresses triple-negative breast cancer stem cells by targeting KLF5 for degradation. Cell Discov. 2017;3:17010. 28480051 10.1038/celldisc.2017.10 PMC5396048

[69] Melnik S, Dvornikov D, Müller-Decker K, Depner S, Stannek P, Meister M, et al. Cancer cell specific inhibition of Wnt/β-catenin signaling by forced intracellular acidification. Cell Discov. 2018;4:37. 29977599 10.1038/s41421-018-0033-2 PMC6028397

[70] Wang Z, Xiao X, Ge R, Li J, Johnson CW, Rassoulian C, et al. Metformin inhibits the proliferation of benign prostatic epithelial cells. PLoS ONE. 2017;12:e0173335. 28253329 10.1371/journal.pone.0173335 PMC5333882

