# Supplementary material for: TNF-α modulates cell proliferation via SOX4/TGF-β/Smad signaling in benign prostatic hyperplasia
Source: Cell Death Dis. 2025 Jul 1;16(1):472. doi: 10.1038/s41419-025-07783-x (PMC12217184; doi:10.1038/s41419-025-07783-x)
Supplement: Supplementary file 1 — Supplementary Information [file 41419_2025_7783_MOESM1_ESM.docx]

**Supplementary Information for**

**TNF-α modulates cell proliferation via SOX4/TGF-β/Smad signaling in benign prostatic hyperplasia**

**Authors:**

Jinze Li^1,2*^, Bo Chen^1*^, Yin Huang^1*^, Xinyang Liao^1^, Jia You^3^, Zeyu Chen^1^, Shu Ning^4^, Asmaa Reda^5,6^, Junwei Zhao^6^, Biao Ran^1^, Jingxing Bai^1^, Mengli Zhu^7^, Yan Wang^7^, Hongying Chen^7^, Qiang Wei^1#^, Dehong Cao^1#^, Liangren Liu^1#^

^1^Department of Urology, Institute of Urology, West China Hospital, Sichuan University, Sichuan, China

^2^Department of Urology, People’s Hospital of Deyang City, Affiliated to Chengdu University of Traditional Chinese Medicine, Deyang, Sichuan, China.

^3^Ningbo Clinical Pathological Diagnosis Center, Ningbo, Zhejiang, China.

^4^Department of Urologic Surgery, School of Medicine, University of California Davis, Davis, CA, USA.

^5^Computational Biology and Bioinformatics, Zoology Department, Faculty of Science, Benha University, Benha, Egypt.

^6^Department of Biochemistry and Molecular Medicine, UC Davis NCI-designated Comprehensive Cancer Center, University of California Davis, Sacramento, CA, USA.

^7^Core Facilities of West China Hospital, Sichuan University, Chengdu, Sichuan, China.

***Corresponding author: Liangren Liu**

**Email: liuliangren@scu.edu.cn**

**This PDF file includes:**

**Table S1 to S7**

**Figure S1 to S9**

**Table S1 ShRNA sequences**

| **Targets** | **No.** | **Sequence** |
| --- | --- | --- |
| SOX4 | Sh#1 | CCTTTCTACTTGTCGCTAAAT |
|  | Sh#2 | GGGCAAGCACCTGGCGGAGAA |
|  | Sh#3 | ACGACCCGAGCTGGTGCAAGA |
| Negative Control | Sh#NC | TTCTCCGAACGTGTCACGT |

**Table S2 Primer sequences**

| **Gene** | **Primer sequence (5'-3')** |
| --- | --- |
| IL-1α | F: ATGGCCAAAGTTCGAGACATG |
|  | R: CTACGCCTGGTTTTCCAGTATCTGAAAGTCAGT |
| IL-1β | F: ATGATGGCTTATTACAGTGGCAA |
|  | R: GTCGGAGATTCGTAGCTGGA |
| IL-6 | F: ATGAACTCCTTCTCCACAAGC |
|  | R: CTACATTTGCCGAAGAGCCCTCAGGCTGGACTG |
| IL-8 | F: ATGACTTCCAAGCTGGCCGTG |
|  | R: TTATGAATTCTCAGCCCTCTTCAAAAACTTCTC |
| IL-18 | F: TCTTCATTGACCAAGGAAATCGG |
|  | R: TCCGGGGTGCATTATCTCTAC |
| SOX4 | F: AGCGACAAGATCCCTTTCATTC |
|  | R: CGTTGCCGGACTTCACCTT |
| FN1 | F: CGGTGGCTGTCAGTCAAAG |
|  | R: AAACCTCGGCTTCCTCCATAA |
| COL1A1 | F: GTGCGATGACGTGATCTGTGA |
|  | R: CGGTGGTTTCTTGGTCGGT |
| α-SMA | F: AAAAGACAGCTACGTGGGTGA |
|  | R: GCCATGTTCTATCGGGTACTTC |
| E-cadherin | F: ATTTTTCCCTCGACACCCGAT |
|  | R: TCCCAGGCGTAGACCAAGA |
| N-cadherin | F: TGCGGTACAGTGTAACTGGG |
|  | R: GAAACCGGGCTATCTGCTCG |
| Vimentin | F: AGTCCACTGAGTACCGGAGAC |
|  | R: CATTTCACGCATCTGGCGTTC |
| GAPDH | F: GGAGCGAGATCCCTCCAAAAT |
|  | R: GGCTGTTGTCATACTTCTCATGG |

**Table S3 Primary antibodies used in western blot experiments**

| **Antibody** | **Concentration** | **Corporation** | **Lot number** |
| --- | --- | --- | --- |
| SOX4 | 1:1000  (1 μg/mL) | GeneTex | GTX31613 |
| TGF-β1 | 1:1000   (0.6 μg/mL) | Abcam | ab215715 |
| Smad2 | 1:1000 | CST | #5339 |
| Smad3 | 1:1000 | CST | #9523 |
| p-smad2 | 1:1000 | CST | #18338 |
| p-smad3 | 1:1000 | CST | #9520 |
| Bcl-2 | 1:1000  (0.75 μg/mL) | ABclonal | A19693 |
| Bax | 1:1000  (1 μg/mL) | ABclonal | A20227 |
| CDK2 | 1:1000  (1.2 μg/mL) | ABclonal | A0094 |
| CDK4 | 1:1000  (1 μg/mL) | ABclonal | A11136 |
| CDK6 | 1:1000 | Zen Bio | R23891 |
| Cyclin A1/A2 | 1:1000  (1.5 μg/mL) | ABclonal | A2635 |
| Cyclin D1 | 1:1000  (0.5 μg/mL) | ABclonal | A19038 |
| Cyclin E2 | 1:1000  (0.6 μg/mL) | ABclonal | A9305 |
| E-Cadherin | 1:1000  (1 μg/mL) | ABclonal | A22333 |
| N-Cadherin | 1:1000  (0.25 μg/mL) | ABclonal | A19083 |
| Vimentin | 1:1000  (1 μg/mL) | ABclonal | A19607 |
| Fibronectin | 1:1000  (0.17 μg/mL) | ABclonal | A16678 |
| COL1A1 | 1:1000  (0.6 μg/mL) | Zen Bio | R26615 |
| α-SMA | 1:1000  (0.2 μg/mL) | Zen Bio | R380653 |
| Vinculin | 1:1000  (1 μg/mL) | ABclonal | A23468/A2752 |
| GAPDH | 1:5000  (1 μg/mL) | Proteintech group | 60004-1-lg |

**Table S4 Antibodies used in immunohistochemistry and immunofluorescence staining**

| **Antibody** | **Concentration** | **Corporation** | **Lot number** |
| --- | --- | --- | --- |
| SOX4 for IF | 1:300  (3.3 μg/mL) | HuaBio | ER1916-97 |
| SOX4 for IHC | 1:200  (5 μg/mL) | GeneTex | GTX31613 |
| TGF-β1 | 1:200 | Abcam | ab215715 |
| E-Cadherin | 1:200  (5 μg/mL) | ABclonal | A22333 |
| N-Cadherin | 1:200  (1.25 μg/mL) | ABclonal | A19083 |
| Vimentin | 1:200  (5 μg/mL) | ABclonal | A19607 |
| Fibronectin | 1:200  (0.85 μg/mL) | ABclonal | A16678 |
| α-SMA | 1:300  (1 μg/mL) | Zen Bio | R380653 |
| Ki-67 | 1:200 | Abcam | ab15580 |

**Table S5 The clinical data of BPH patients**

| **Variables** | **Mean** | **Standard deviation** |
| --- | --- | --- |
| Age (year) | 67.15 | 8.83 |
| BMI (kg/m^2^) | 23.30 | 2.82 |
| tPSA (ng/mL) | 5.28 | 3.76 |
| PV (mL) | 54.77 | 21.36 |
| IPSS score | 22.20 | 3.15 |
| Qmax (mL/s) | 11.33 | 3.01 |
| PVR (mL) | 69.95 | 64.02 |

Note: Weight (kg) and height (cm) were measured by a trained nurse using a standardized protocol. Body mass index (BMI) was as weight divided by the square of the height (kg/m2). Prostate volume (PV) was measured by transrectal ultrasound and is calculated using the following formula for an elliptic volume (height × width × length × π/6), and the International prostate Symptom Score (IPSS) was obtained by filling out the relevant questionnaire. Post-void residual urine volume (PVR) was also measured by ultrasound. The maximum urinary flow rate (Qmax) was evaluated by uroflowmetry, and the voided volumes had to be > 150 ml to avoid bias. Blood samples were drawn from participants after a 12-h fast, and serum total prostate-specific antigen (tPSA) was recorded.

**Table S6 Correlation between serum TNF-α concentration and clinical indicators in patients with BPH**

| **Variables** | ***r*** | ***P*** |
| --- | --- | --- |
| Age | -0.020 | 0.912 |
| BMI | -0.019 | 0.919 |
| tPSA | 0.193 | 0.289 |
| PV | 0.5533 | 0.001 |
| IPSS score | 0.5224 | 0.002 |
| Qmax | 0.346 | 0.052 |
| PVR | -0.000 | 0.996 |

**Table S7 Rats’ body weight and prostate parameters.**

| **Group** | **Body weight (g)** | **Prostate weight (g)** | **Prostate index (PI)** |
| --- | --- | --- | --- |
| Control | 497.94 ± 29.98 | 0.51 ± 0.76 | 1.02 ± 0.12 |
| BPH | 415.92 ± 27.51^a^ | 0.82 ± 0.12^a^ | 1.98 ± 0.28^a^ |
| BPH + Met | 423.48 ± 29.80^b^ | 0.59 ± 0.08^b^ | 1.40 ± 0.13^b^ |

Note: The values are described as mean ± SD (n = 5 animals/group). ^a^*p* < 0.05 vs. control. ^b^*p* < 0.05 vs. BPH. Different letters in a column represent the statistical difference (*p* < 0.05).


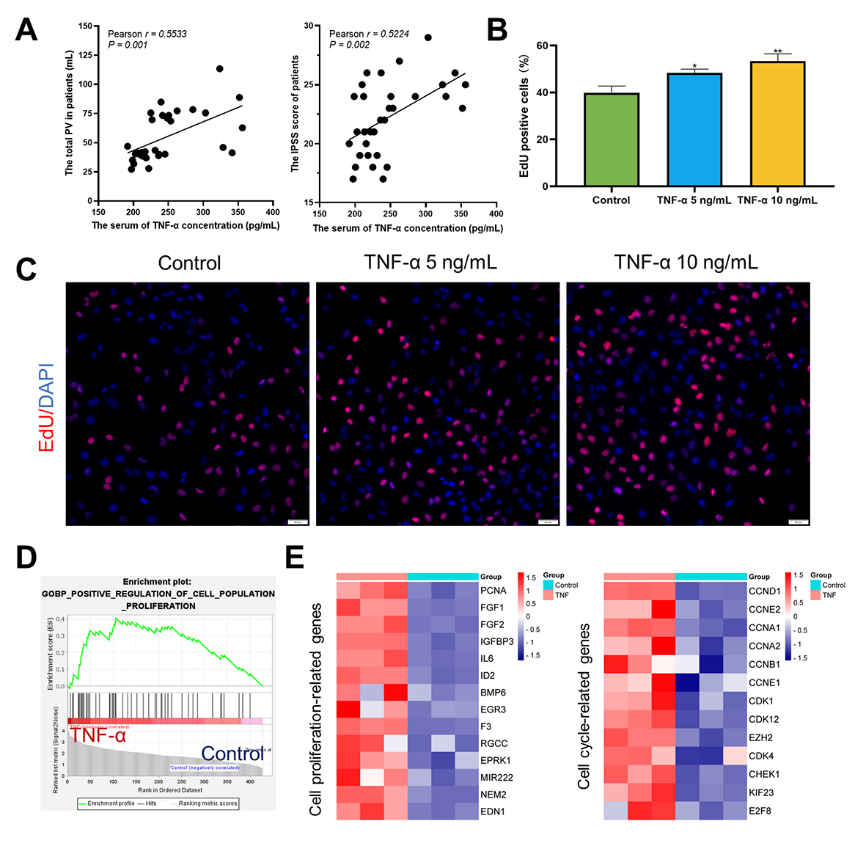


**Figure S1. TNF-α expression is positively correlated with clinical symptom severity and enhances the proliferation of WPMY-1 cells**

**A.** The correlation between TNF-α levels in serum and prostate volume, and IPSS score in BPH patients. **B-C.** WPMY-1 cells were plated in 96-well plates (3 × 10^3^ cells/well) and treated with TNF-α (5 ng/mL and 10 ng/mL) for 3 days. EdU assay was used to detect the cell proliferation viability. **D.** Enrichment plot of GSEA analysis for the upregulation of cell proliferation pathways in TNF-α group compared with control group in WPMY-1 cells. **E.** Heatmap clustering the up-regulated genes related with cell proliferation and cell cycle in WPMY-1 cells (***p*<0.05, ***p* < 0.01).


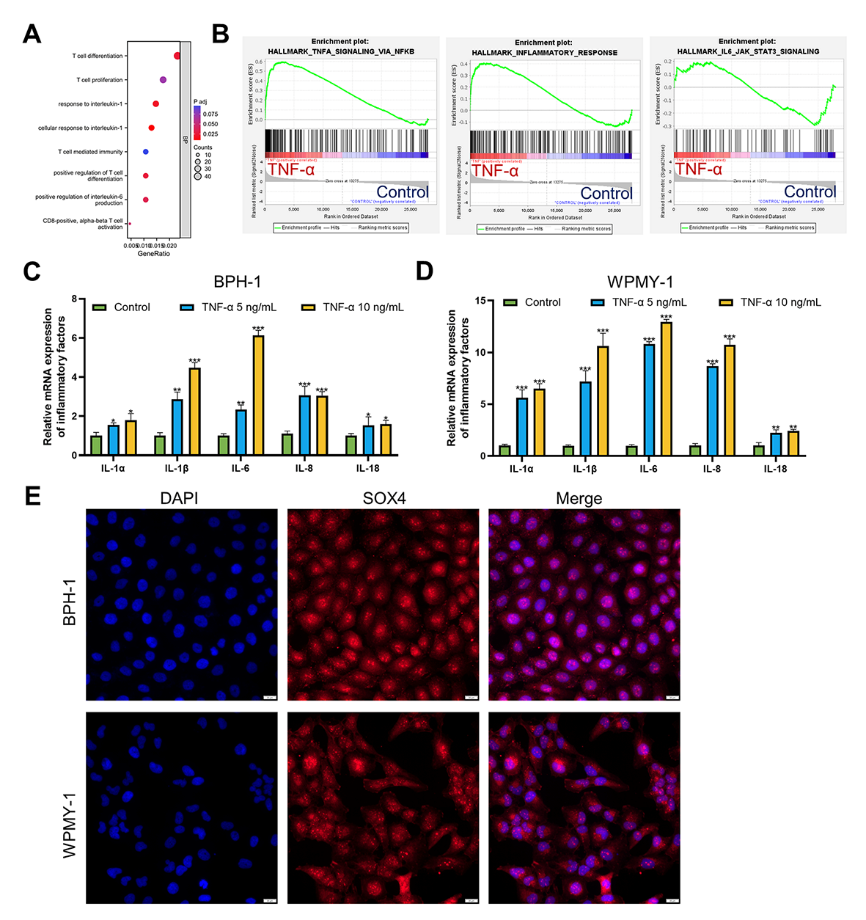


**Figure S2. TNF-α regulates inflammatory related gene programs in BPH cells**

**A.** GO analysis showing inflammatory related pathways in WPMY-1 cells treated with TNF-α. **B.** Enrichment plots of GSEA analyses for inflammatory related pathways in TNF-α group compared with control group. **C-D.** RT-PCR was used to test the mRNA levels of inflammation related genes in BPH-1 and WPMY-1 cells treated with TNF-α. **E.** Cell immunofluorescence was used to test the expression and location of SOX4 protein in BPH-1 and WPMY-1 cells (* *p*<0.05, ** *p*<0.01, ****p* < 0.001).


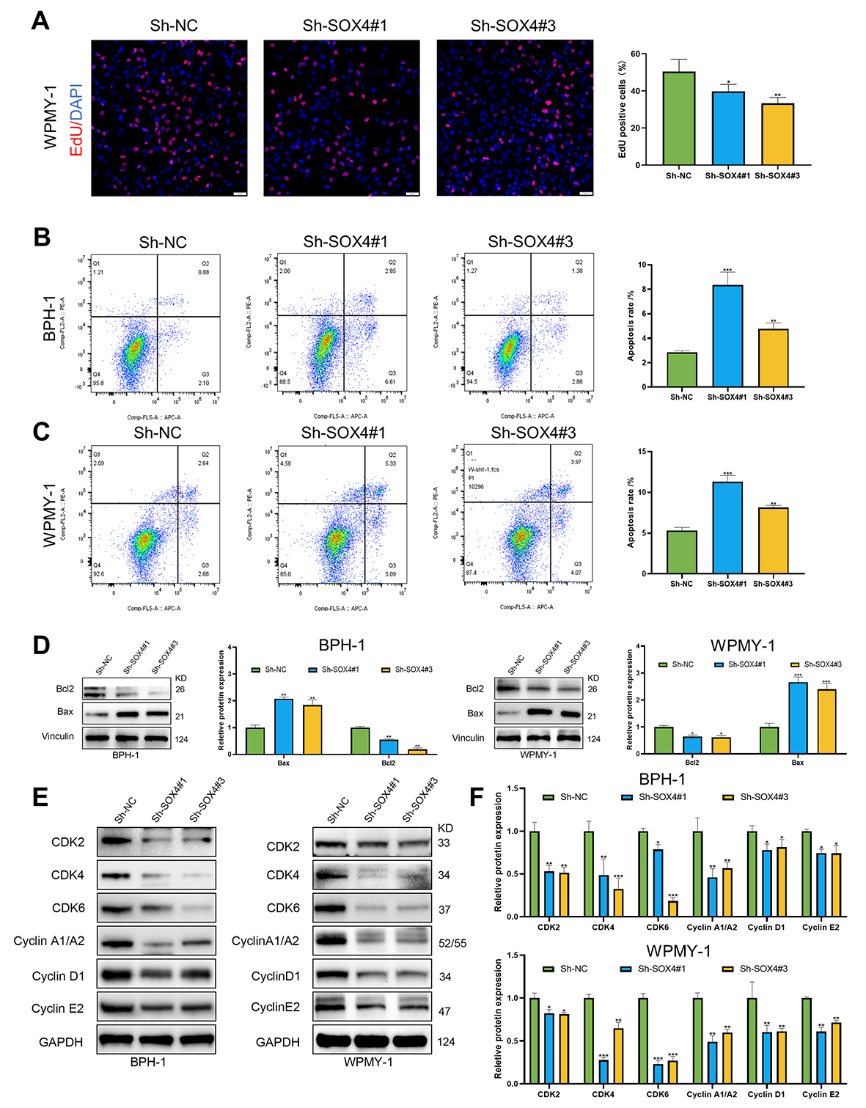


**Figure S3. Effects of SOX4 knockdown on proliferation, apoptosis and expression of related proteins in BPH cells**

**A.** WPMY-1 cells were plated in 96-well plates (3 × 10^3^ cells/well) and infected with Sh-NC or Sh-SOX4 lentivirus for 3 days. EdU assay was used to detect the cell proliferation activity. **B-C**. BPH-1 and WPMY-1 cells were plated in 6-well plates (2 × 10^5^ cells/well). Flow cytometry was used to test the cells apoptosis of BPH-1 and WPMY-1 cells infected with Sh-NC or Sh-SOX4 lentivirus for 3 days. **D**. BPH-1 and WPMY-1 cells infected with Sh-NC or Sh-SOX4 lentivirus for 3 days. Then, whole cell lysates of BPH-1 and WPMY-1 cells were harvested for testing Bcl2 and Bax proteins expression levels. **F-F**. BPH-1 and WPMY-1 cells infected with Sh-NC or Sh-SOX4 lentivirus for 3 days. Then, whole cell lysates of BPH-1 and WPMY-1 cells were harvested for testing CDK2 and other proteins expression levels (* *p*<0.05, ** *p*<0.01, ****p* < 0.001).


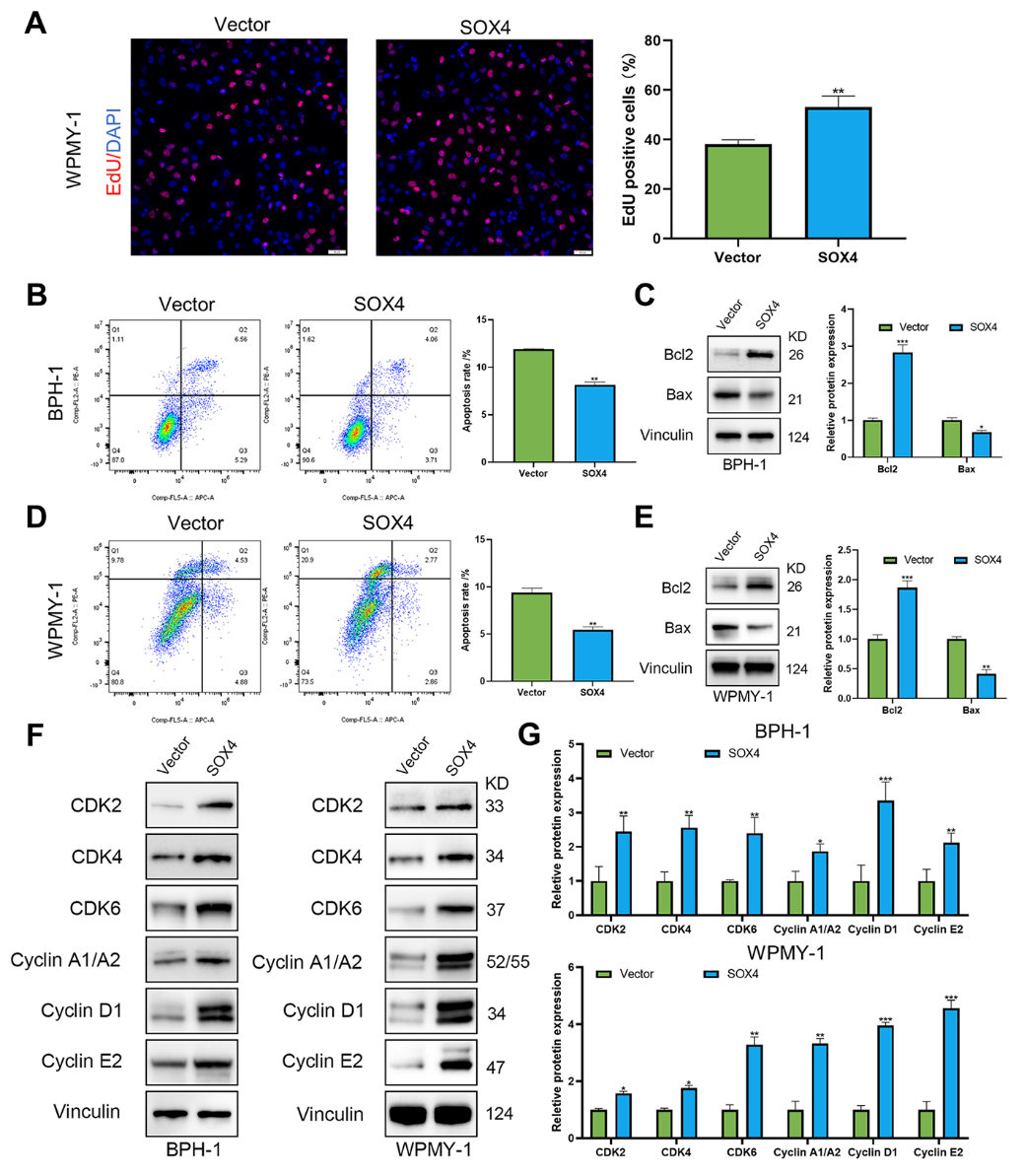


**Figure S4. Effects of SOX4 overexpression on proliferation, apoptosis and expression of related proteins in BPH cells**

**A.** WPMY-1 cells were plated in 96-well plates (3 × 10^3^ cells/well) and infected with vector or SOX4 lentivirus for 3 days. EdU assay was used to detect the cell proliferation activity. **B-E.** BPH-1 and WPMY-1 cells were plated in 6-well plates (2 × 10^5^ cells/well). Flow cytometry was used to test the cells apoptosis of BPH-1 and WPMY-1 cells infected with vector or SOX4 lentivirus for 3 days. WB was used to test the protein levels of apoptosis markers in BPH-1 and WPMY-1 cells infected with vector or SOX4 lentivirus for 3 days. **F-****G**. BPH-1 and WPMY-1 cells infected with vector or SOX4 lentivirus for 3 days. Then, whole cell lysates of BPH-1 and WPMY-1 cells were harvested for testing CDK2 and other proteins expression levels (* *p*<0.05, ** *p*<0.01, ****p* < 0.001).


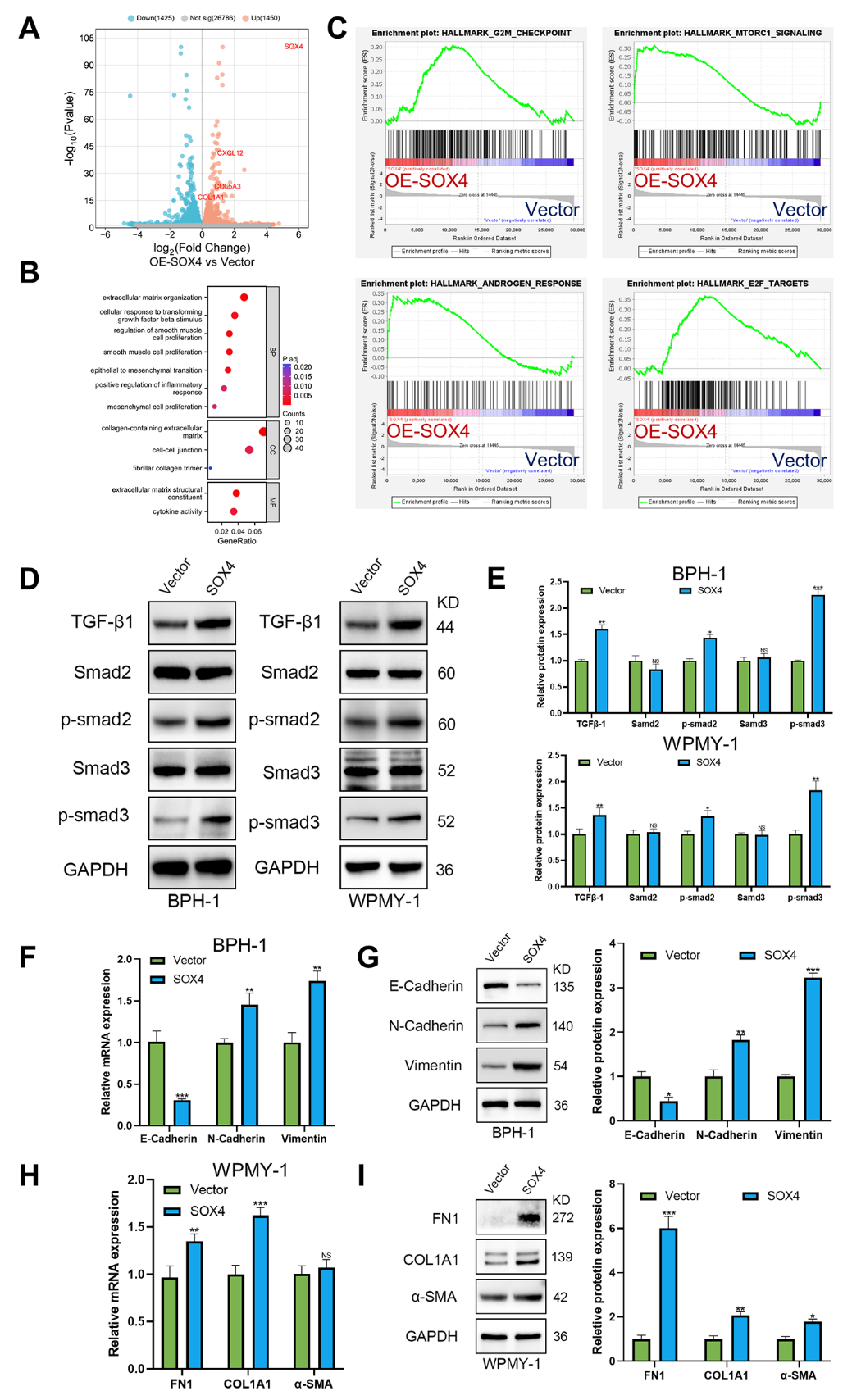


**Figure S5.** **Overexpression of SOX4 regulates TGF-β/Smad signaling pathway and its downstream genes**

**A.** Valcano plot showing the differentiated expressed genes in WPMY-1 cells overexpressing SOX4. **B.** Enrichment plots of GSEA analyses for significant pathways in overexpression SOX4 group compared with vector group in WPMY-1 cells. **C.** GO analysis showing pathways down/up-regulated in WPMY-1 cells overexpressing SOX4. **D-E.** WB analysis of TGF-β/Smad pathway protein expression in BPH-1 and WPMY-1 cells infected with vector or overexpression SOX4 lentivirus for 3 days. **F-G.** RT-PCR and WB analysis of EMT marker expression in BPH-1 cells infected with vector or overexpression SOX4 lentivirus for 3 days, respectively. **H-I.** RT- PCR and WB analysis of fibrosis marker expression in WPMY-1 cells infected with vector or overexpression SOX4 lentivirus for 3 days (* *p*<0.05, ** *p*<0.01, *** *p*< 0.001, ns: not significant).


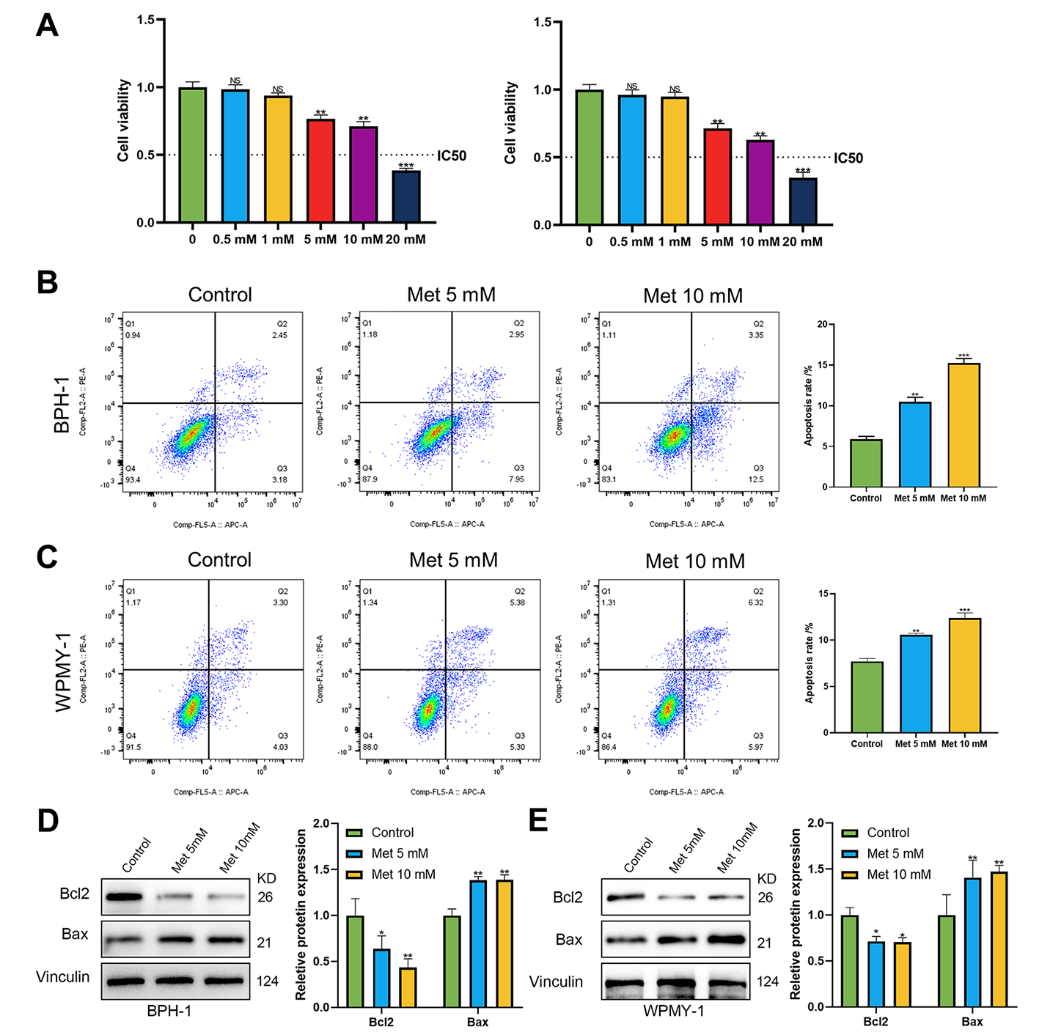


**Figure S6. Met maybe served as a novel inhibitor of SOX4 for improving BPH treatment**

**A.** BPH-1 and WPMY-1 cells were plated in 96-well plates (3 × 10^3^ cells/well) and cultured overnight. Subsequently, the cells were treated with Met at concentrations of 0, 0.5, 1, 5, 10, and 20 mM for 3 days. Cell viability was then assessed using the CCK-8 assay. **B-C.** BPH-1 and WPMY-1 cells were plated in 6-well plates (2 × 10^5^ cells/well) overnight. Then, BPH-1 and WPMY-1 cells treated with Met (0, 5, and 10 mM) for 3 days, and harvested for cell apoptosis test via flow cytometry. **C-D**. WB analysis of apoptosis related proteins (Bcl2 and Bax) expression in BPH-1 and WPMY-1 cells treated with Met (0, 5, and 10 mM) for 3 days (* *p*<0.05, ** *p*<0.01, ****P* < 0.001).


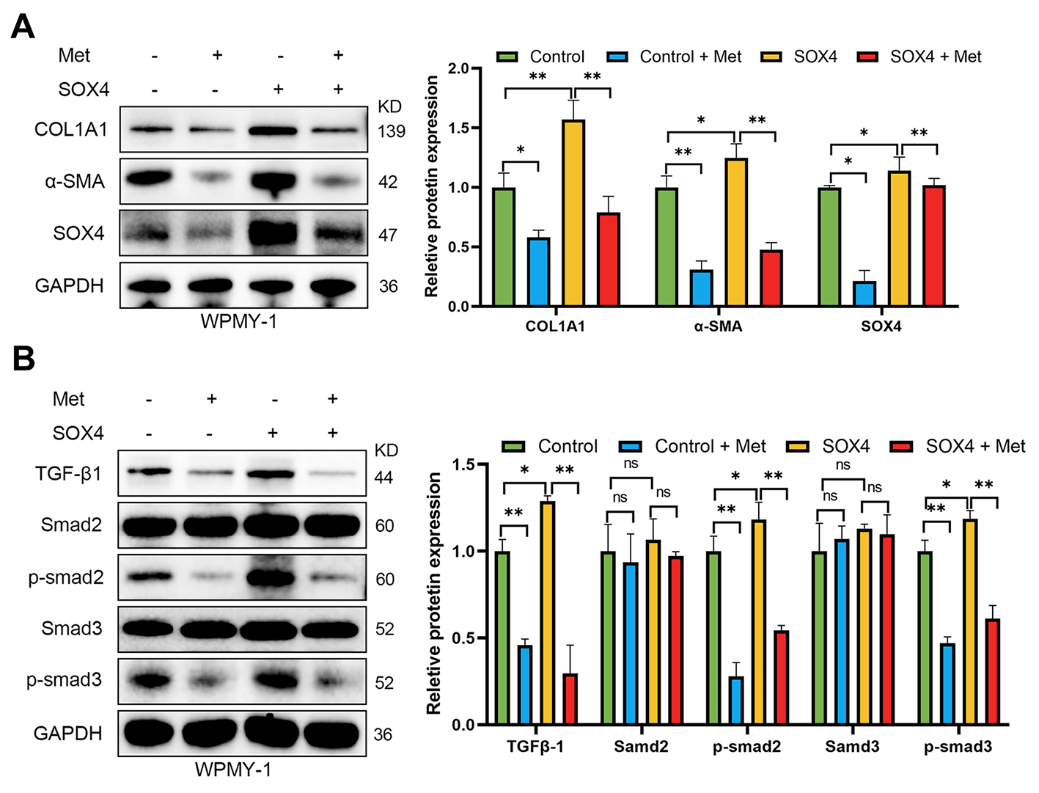


**Figure S7. Overexpression of SOX4 partially reverse the inhibitory effects of Met on the TGF-β/Samd signaling pathway and its downstream genes**

**A.** WB analysis of SOX4 and fibrosis marker protein expression in WPMY-1 cells treated with Vector, Vector combined with Met, overexpression SOX4, or overexpression SOX4 combined with Met for 3 days. **B.** WB analysis of TGF-β/Smad pathway protein expression in WPMY-1 cells treated with Vector, Vector combined with Met, overexpression SOX4, or overexpression SOX4 combined with Met for 3 days (* *p*<0.05, ** *p*<0.01, ns: not significant).


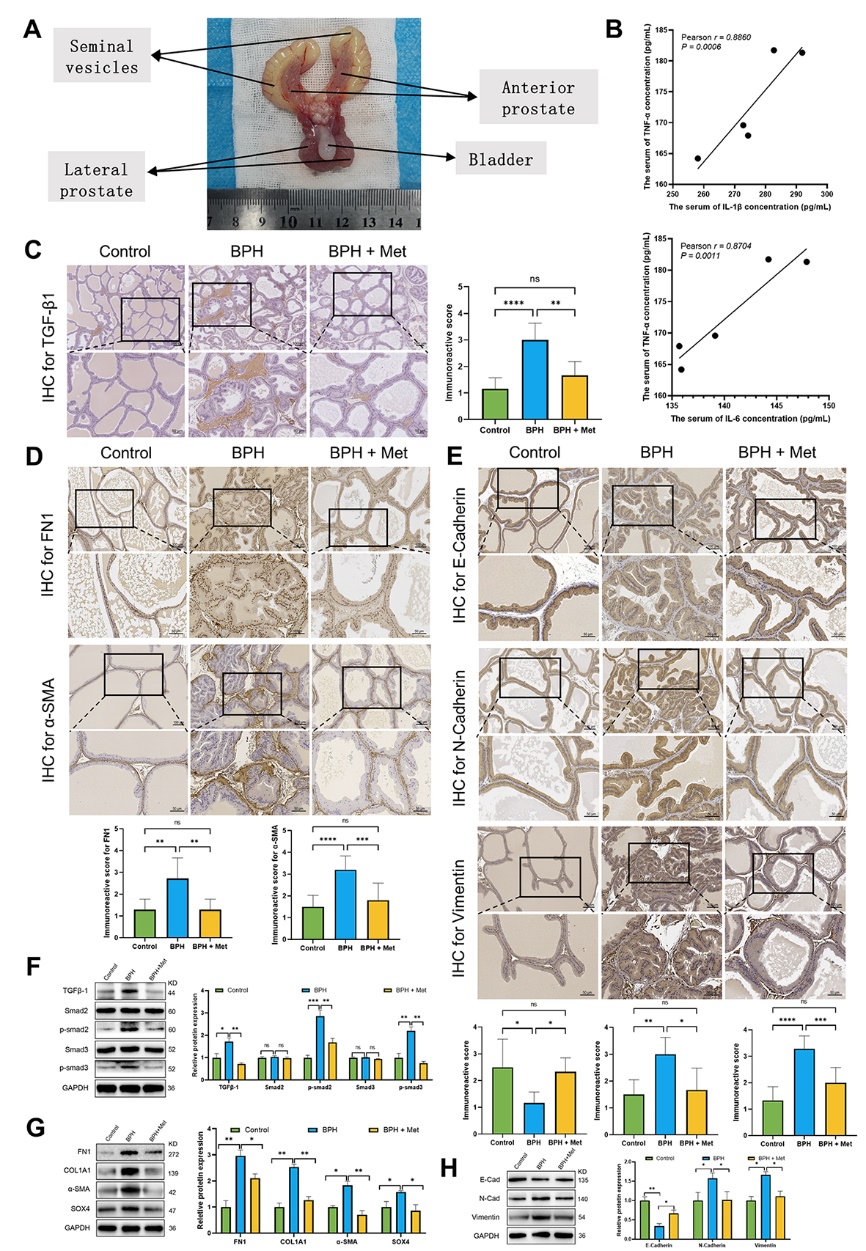


**Figure S8. Met inhibits the SOX4/TGF-β/Smad signaling axis *in vivo***

**A.** Prostate picture showing the anatomy structure of rat prostate. **B.** Correlation analysis between TNF-α and IL-1β, and IL-6 in serum. **C.** Representative IHC staining of TGF-β1 in prostate samples from the control, BPH, and BPH combined with Met groups. **D.** Representative IHC staining of fibrosis markers (FN1 and α-SMA) in prostate samples from the control, BPH, and BPH combined with Met groups. **E.** Representative IHC staining of EMT markers (E-Cadherin, N-Cadherin, and Vimentin) in prostate samples from the control, BPH, and BPH combined with Met groups. **F-H.** Whole lysates of prostate were harvested in control, BPH, and BPH combined with Met group. Then, WB analysis was performed to evaluate the expression of TGF-β/Smad pathway, fibrosis, and EMT marker proteins in the rat model (* *p*<0.05, ** *p*<0.01, *** *p*<0.001, **** *p*<0.0001, ns: not significant).


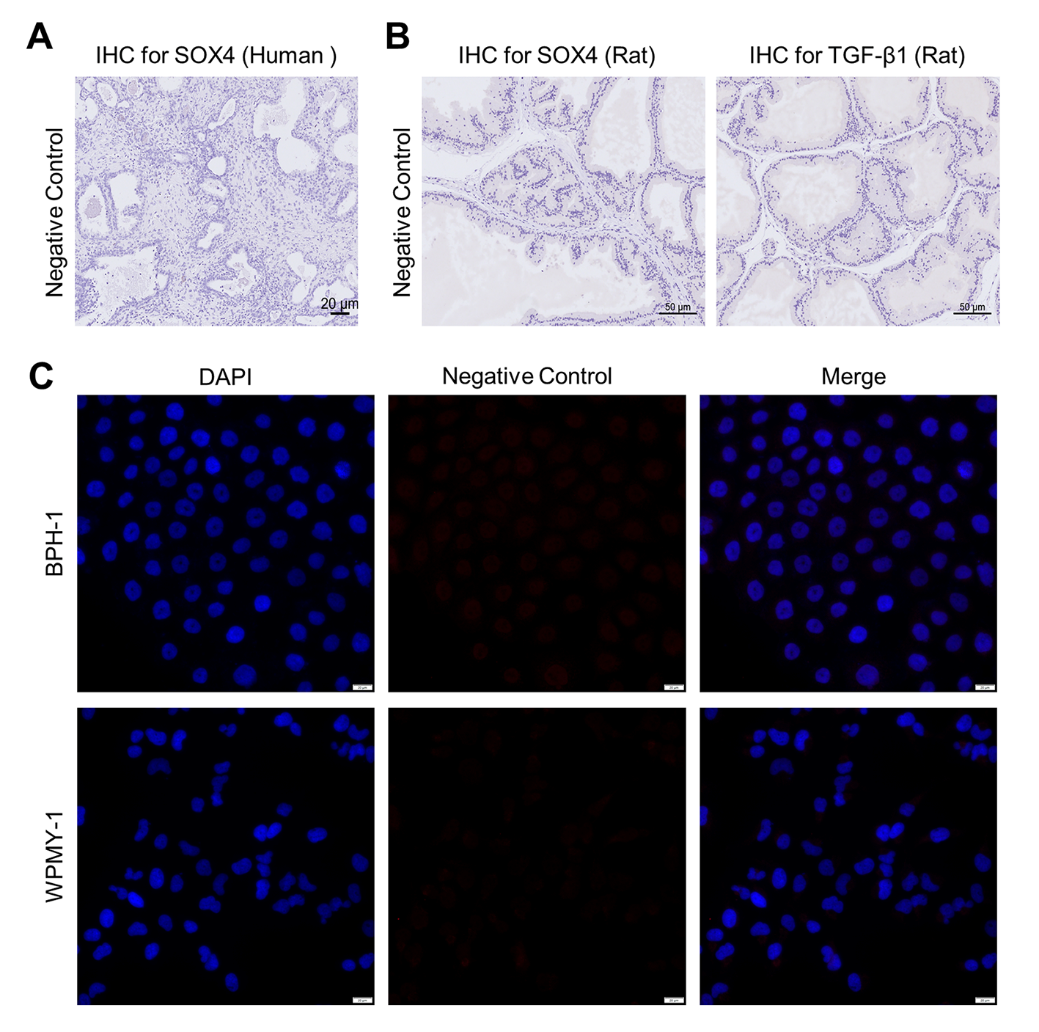


**Figure S9. Negative controls for IHC and immunofluorescence staining**

**A.** Representative IHC staining of a SOX4 negative control from human prostate sample. **B.** Representative IHC staining of SOX4 and TGF-β1 negative controls from Rat prostate sample. **C.** Immunofluorescence staining of SOX4 negative controls in BPH-1 and WPMY-1 cells.
