# Supplementary figures and images for: TNF-α modulates cell proliferation via SOX4/TGF-β/Smad signaling in benign prostatic hyperplasia
Source: Cell Death Dis. 2025 Jul 1;16(1):472. doi: 10.1038/s41419-025-07783-x (PMC12217184; doi:10.1038/s41419-025-07783-x)

**Original images of western blot gel**

**
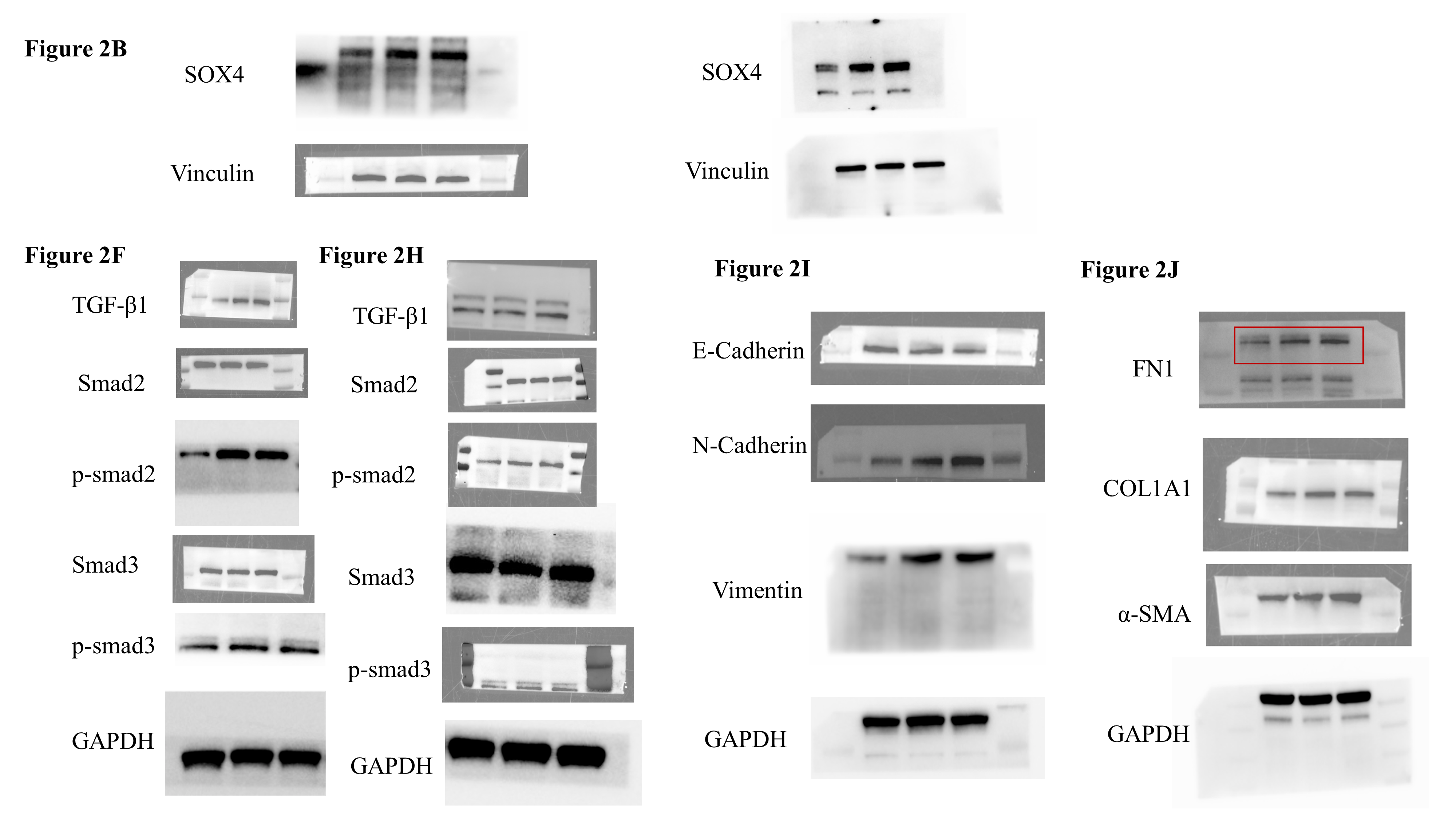
**


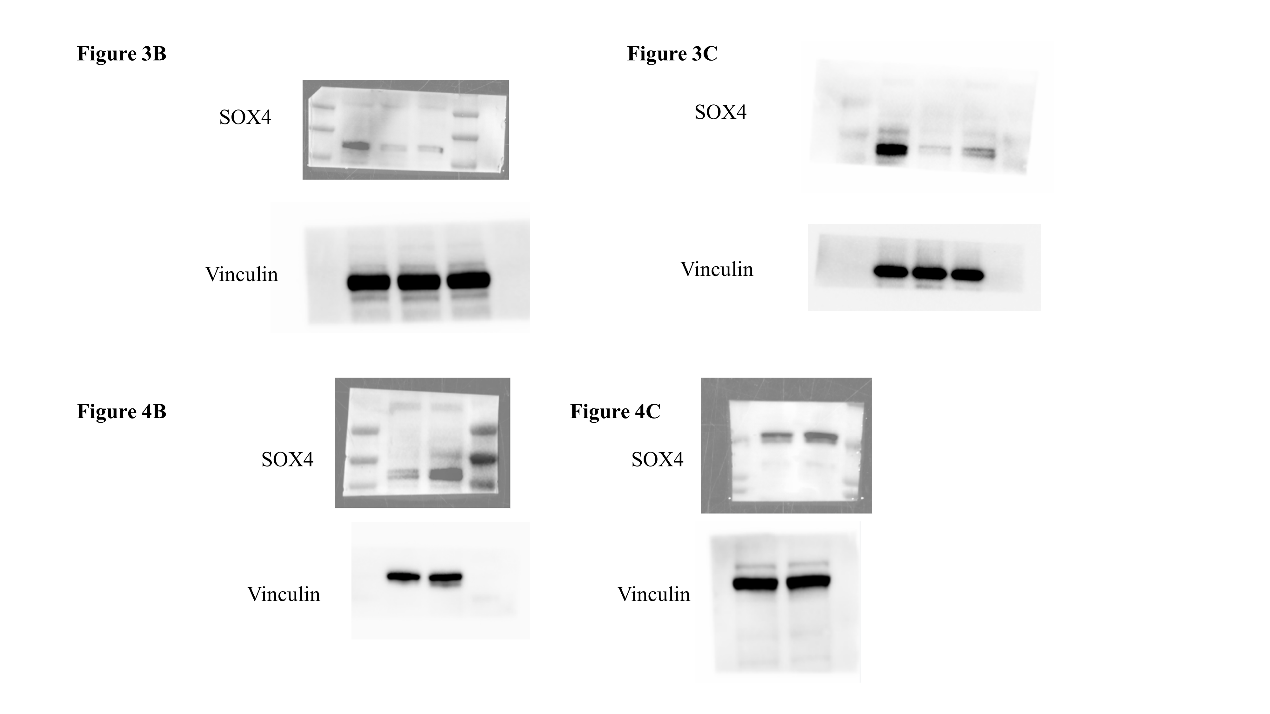


**
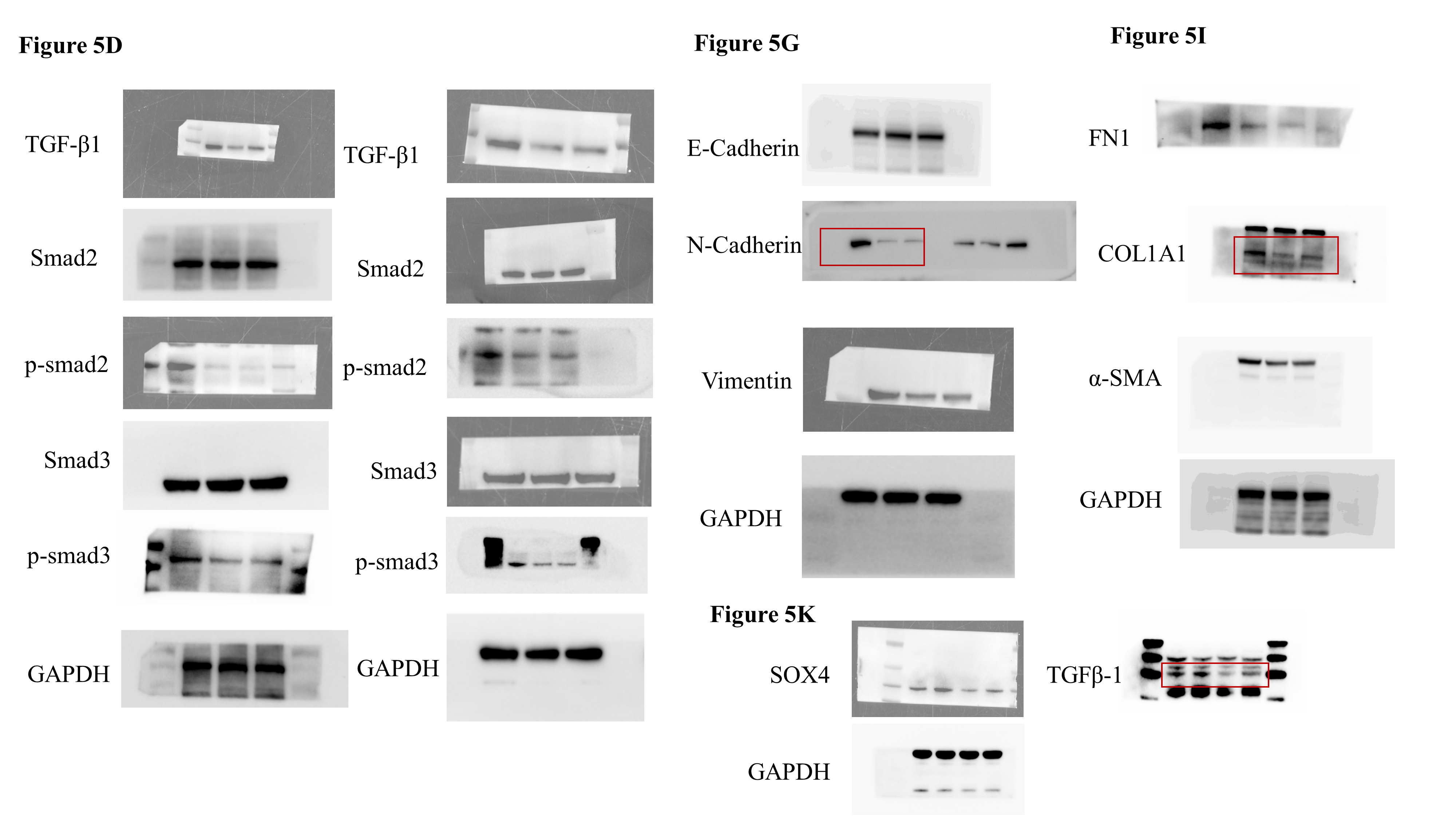
**

**
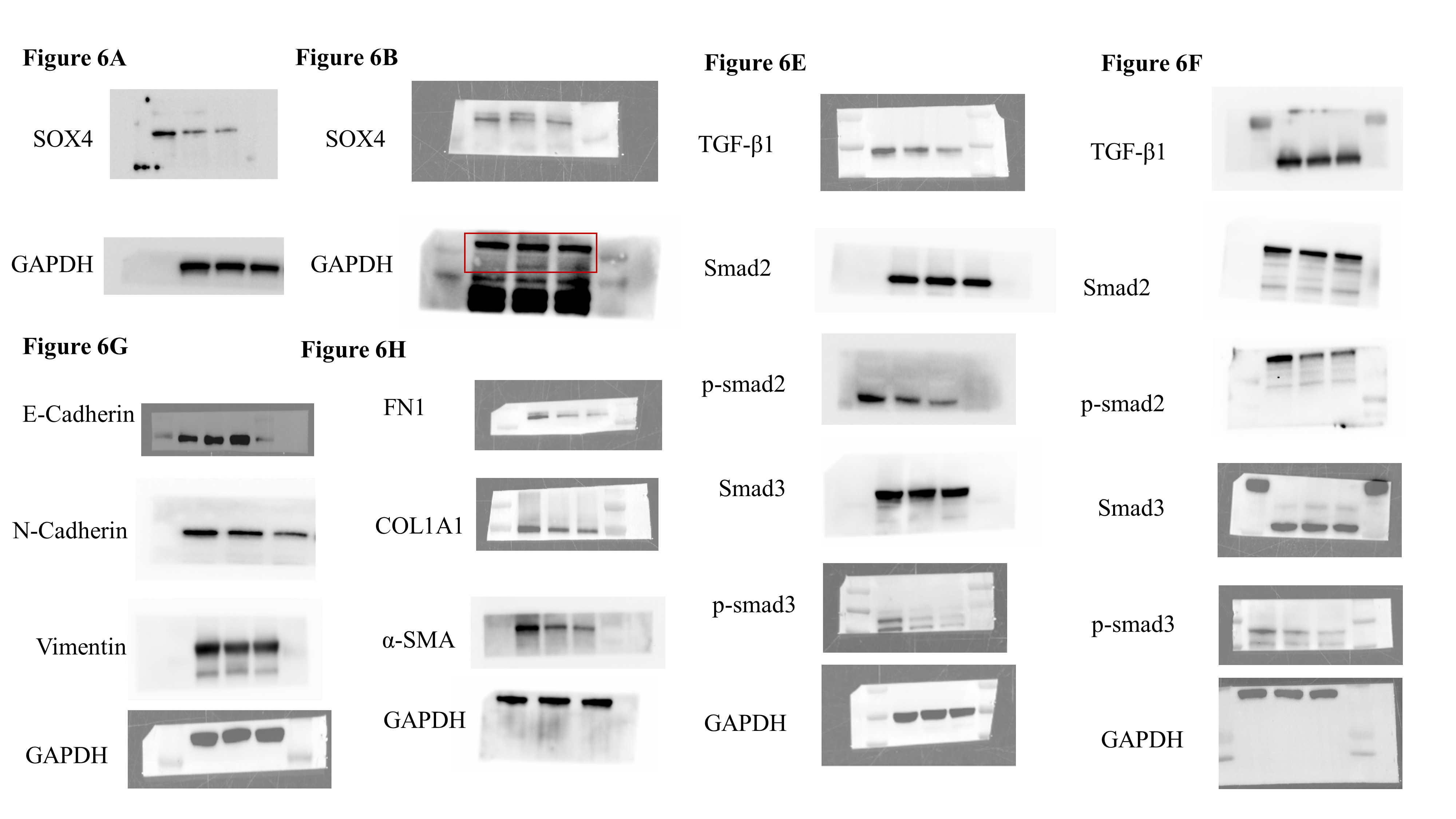
**

Supplement: Supplementary file 2 — Original images of western blot gel [file 41419_2025_7783_MOESM2_ESM.docx]
